# An implementation of integrated information theory in resting-state fMRI

**DOI:** 10.1038/s42003-023-05063-y

**Published:** 2023-07-05

**Authors:** Idan E. Nemirovsky, Nicholas J. M. Popiel, Jorge Rudas, Matthew Caius, Lorina Naci, Nicholas D. Schiff, Adrian M. Owen, Andrea Soddu

**Affiliations:** 1 Western Institute for Neuroscience, Department of Physics and Astronomy, University of Western Ontario, 1151 Richmond St, London, ON N6A 3K7 Canada; 2 Cavendish Laboratory, University of Cambridge, Cambridge, CB3 0HE United Kingdom; 3 Institute of Biotechnology, Universidad Nacional de Colombia, Cra 45 Bogotá, Colombia; 4 Department of Medical Biophysics, University of Western Ontario, 1151 Richmond St, London, ON N6A 3K7 Canada; 5 Trinity College Institute of Neuroscience, Trinity College Dublin, Dublin 2, Ireland; 6 Feil Family Brain Mind Research Institute, Weill Cornell Medical College, New York, NY 10065 USA; 7 Department of Physiology and Pharmacology and Department of Psychology, University of Western Ontario, 1151 Richmond St, London, ON N6A 3K7 Canada

**Keywords:** Consciousness, Network models

## Abstract

Integrated Information Theory was developed to explain and quantify consciousness, arguing that conscious systems consist of elements that are integrated through their causal properties. This study presents an implementation of Integrated Information Theory 3.0, the latest version of this framework, to functional MRI data. Data were acquired from 17 healthy subjects who underwent sedation with propofol, a short-acting anaesthetic. Using the PyPhi software package, we systematically analyze how Φ^max^, a measure of integrated information, is modulated by the sedative in different resting-state networks. We compare Φ^max^ to other proposed measures of conscious level, including the previous version of integrated information, Granger causality, and correlation-based functional connectivity. Our results indicate that Φ^max^ presents a variety of sedative-induced behaviours for different networks. Notably, changes to Φ^max^ closely reflect changes to subjects’ conscious level in the frontoparietal and dorsal attention networks, which are responsible for higher-order cognitive functions. In conclusion, our findings present important insight into different measures of conscious level that will be useful in future implementations to functional MRI and other forms of neuroimaging.

## Abstract (web summary)

Resting-state fMRI data in conditions of wakefulness and anaesthesia was analyzed using integrated information theory and its key measure, Φmax, which presents a variety of sedative-induced behaviours across different cortical networks.

## Introduction

Consciousness is a remarkably complex concept that continues to be a subject of great debate in the neuroscientific community.^1^ An individual would typically be described as conscious if they demonstrate wakefulness and awareness.^2^ However, neuroscientific literature continues to show that consciousness transcends the mere absence or presence of these behavioral traits.^3,4^

While many questions remain to be answered, our knowledge about conscious phenomena has been greatly improved by the use of advanced neuroimaging tools. One such tool is functional magnetic resonance imaging (fMRI), which allows for observation of cortical activity in spatial and temporal domains.^5^ Activity measured with fMRI represents blood flow patterns in the form of the blood-oxygen-level-dependent (BOLD) signal.^6–8^ Although fMRI studies were initially designed to have subjects perform specific tasks, it has become increasingly common to measure spontaneous brain activity without overt stimulation. Such acquisition is called resting-state fMRI, and studies involving it have identified a series of Resting State Networks (RSNs), which are collections of cortical regions that behave in synchrony and represent the brain’s functional organization at rest.^7,8^

In both task-based and resting-state studies, fMRI and other neuroimaging tools have been used to establish neural correlates of consciousness, which are aspects of brain activity that correspond to conscious processes.^9^ While these correlates are used to explain specific conscious percepts (i.e., reaction to stimuli, emotions, thoughts), attempts have also been made to explain consciousness more generally, with the aim of accounting for a wide range of conscious phenomena.

In keeping with these developments, the present study is focused on integrated information theory (IIT) and its principal metric, integrated information (Φ), which presents a thorough interpretation of consciousness and attempts to quantify it for physical systems consisting of interacting elements, such as the brain.^10–12^ IIT begins by analyzing the phenomenological properties of consciousness, which are used to deduce a set of axioms about the nature of a conscious experience. The fundamental property of consciousness is intrinsic existence, meaning that a system is conscious from its own perspective, regardless of how it may be viewed externally.^12^ Information describes the specific features of a conscious experience, and in accordance with the first axiom, must be an intrinsic quantity.^13^ The distinction between intrinsic information and measures of extrinsic information (i.e., Shannon entropy) is crucial to IIT, which strictly pertains to the former type.

These ideas build up to the principal argument of IIT, which is that consciousness arises in a system that generates more information as an integrated whole than that which is generated by the sum of its parts. In other words, the interactions between a system’s elements, which are governed by its mechanisms, yield a level of information that is higher than when the system is reduced to its individual components.^12,14^ This property is called irreducibility and is quantified using Φ, which is computed by partitioning the system (i.e., breaking connections between its elements) and measuring the subsequent loss of information.

So far, three versions of IIT have been introduced. The initial framework (IIT 1.0) was limited to stationary systems^10^, while the second version (IIT 2.0) extended the theory to dynamic systems that evolve over time.^11,15^ The latest version, IIT 3.0, introduced a series of theoretical advancements and formulated the most computationally intensive variant of Φ, known as the maximally integrated conceptual information (Φ^max^).^12^ Fundamentally, IIT 3.0 treats information as causation; starting with how a system’s elements interact (i.e., the system’s mechanisms), information is computed by relating the system’s present state to all of its possible causes and effects. On the other hand, IIT 2.0 computes the mutual information between the system’s past and present states, which does not involve the same level of consideration for its causal mechanisms.^16,17^

The theoretical and quantitative aspects proposed by IIT are promising, and some progress has been made to incorporate measures derived from IIT 2.0 to fMRI and EEG data. In a task-based fMRI study that presented subjects with a movie clip followed by visual noise as a control, Boly et al. found that Φ dropped significantly during the control paradigm, meaning it could effectively capture the integration of meaningful stimuli.^18^ Similar measures were applied in an fMRI study on functionally-split properties of brain activity, which shed light on the possibility of multiple streams of consciousness when individuals perform simultaneous tasks.^19^ Finally, IIT was also applied in a study involving high-density EEG recordings in which the authors used parameters derived from Φ to differentiate different states of consciousness.^20^

Despite these developments, existing literature on IIT remains largely theoretical, and more efforts are needed to develop procedures that can be used to extract empirical measures of Φ. This is especially the case for the latest version of IIT, as there are currently no published works that attempt to quantify Φ^max^ for neuroimaging or EEG data. While IIT 3.0 involves a considerably more rigorous mathematical formulation than previous versions, Φ^max^ remains limited to discrete Markovian systems consisting of binary elements, which presents a limitation in its suitability to empirical imaging data. Nevertheless, we believe that a preliminary implementation is possible with the tools currently available. This work hence presents a procedure to quantify Φ^max^ for empirical resting-state fMRI data, which we hope will serve as a foundation for further empirical applications of integrated information theory.

**Fig. 1:**
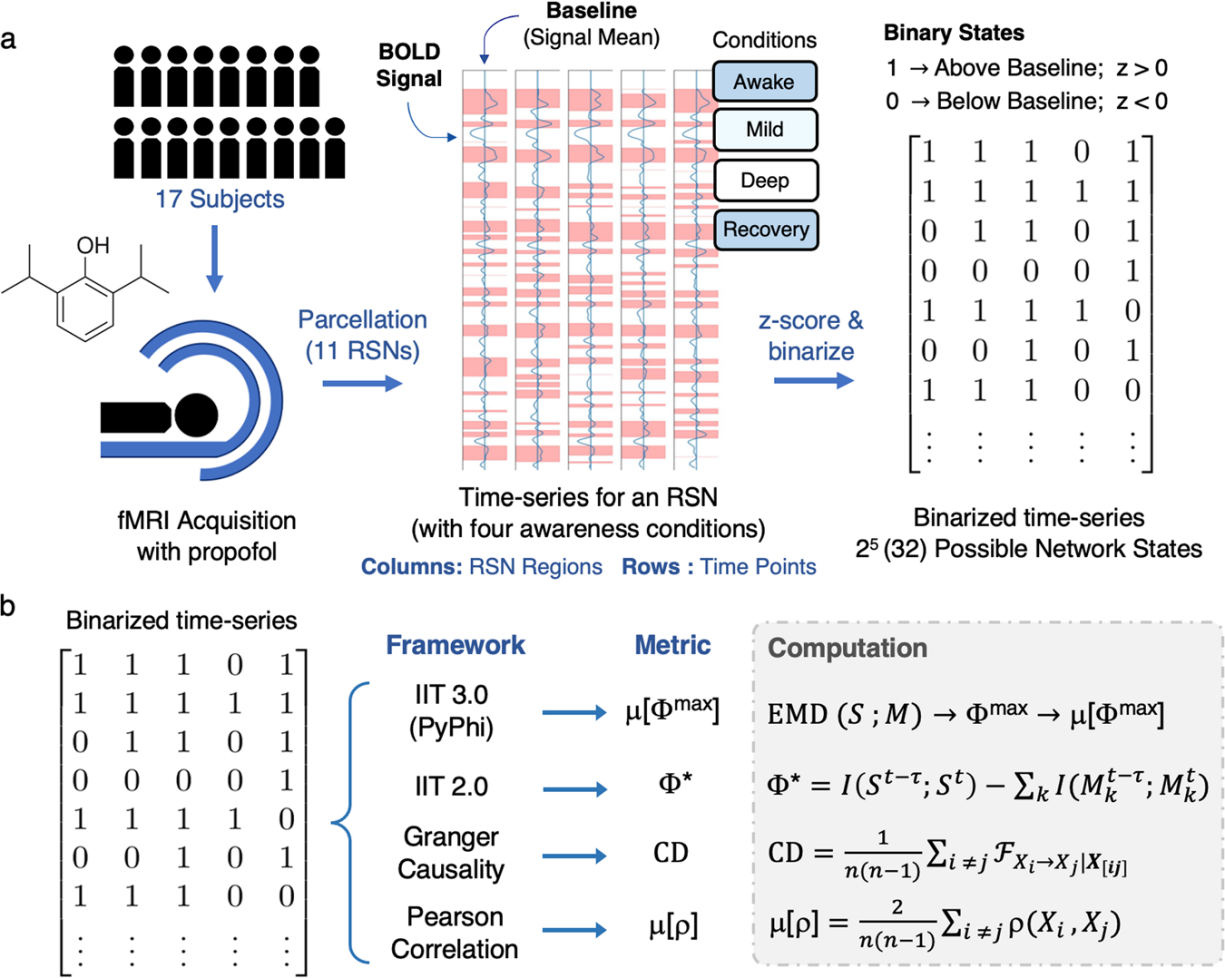
Summary of acquisition, signal processing, and metric extraction. **a** fMRI data were acquired from 17 healthy subjects and propofol was administered to obtain measurements for four conditions of awareness: Awake: no propofol administered, Mild: Ramsey Scale Level 3 (limited responsiveness), Deep: Ramsey Scale Level 5 (no responsiveness), Recovery: administration of anesthetic terminated (see Methods for more details). We then applied a parcellation scheme and obtained 11 RSNs consisting of five regions, each with their own time-series. The time-series of each region was binarized with respect to its own signal mean, which allowed each network to take on 32 (or 2^5^) possible states in any given time point. Note that we concatenated (joined) the time-series from individual subjects to obtain longer signals for each RSN and condition. **b** The metric derived from IIT 3.0 is μ[Φ^max^]: Φ^max^ is computed for a certain state using the earth mover’s distance (${{{{{\rm{EMD}}}}}}$) between the cause-and-effect conceptual space of the system *S* and its partitioned counterparts *M*. We computed Φ^max^ for each state and obtained a weighted average over the time-series. The three additional metrics included in our analysis are: Φ*, integrated information from the decoding perspective, which is computed as the difference between the mutual information *I* of the system’s past and present states (${S}^{t-\tau },{S}^{t}$) and the sum over the mutual information of the partitioned parts (${M}_{k}^{t-\tau },{M}_{k}^{t}$); ${{{{{\rm{CD}}}}}}$, causal density, the mean Granger causality ${{{{{\mathscr{F}}}}}}$ between every possible pair of regions in the network; and μ[ρ], the mean Pearson correlation coefficient ρ between every possible pair of regions in the network.

Resting-state fMRI data were acquired from 17 healthy subjects who underwent sedation with propofol, a short-acting anesthetic. Images were parcellated to obtain 11 RSNs, which were processed to include five regions for reasonable computation times. Measurements of resting-state activity were obtained over four conditions of awareness: awake, mildly sedated, deeply sedated, and recovery. To obtain networks with discrete elements as required for IIT 3.0, each RSN’s time-series were standardized and converted to a binary form. In keeping with the importance of intrinsicality, each region’s threshold was acquired with respect to its own mean signal strength (see Fig. 1a and Methods for more details). The software used to compute Φ^max^ was PyPhi, a Python package developed in accordance with IIT 3.0.^12,21^ Since Φ^max^ is a state-dependent quantity that changes over time, we computed its weighted average over the time-series of each network, which we refer to as μ[Φ^max^].

Our central investigation seeks to determine whether μ[Φ^max^] can yield meaningful quantitative differences across conscious conditions, as well as which RSNs demonstrate these changes. If a measure of cortical activity can serve as a valid marker of consciousness, its value should gradually decrease as subjects transition from wakefulness to deep sedation and increase as the anesthetic wears off during recovery. It was previously reported that propofol-induced anesthesia predominantly suppresses RSNs with frontal and prefrontal regions, which are associated with higher-order functions; on the other hand, the effects on sensory networks are less pronounced.^22,23^ Accordingly, we expect μ[Φ^max^] to present a variety of anesthetic-induced variations across the networks, and our discussion will relate these results to existing literature on propofol.

Since this study is an experimentation with IIT 3.0, we also consider how μ[Φ^max^] compares with other metrics that may reflect conscious level. Therefore, we introduce three additional measures throughout our analysis, which are: 1) Φ*; integrated information from the decoding perspective, a measure derived from IIT 2.0^13,24^; 2) Causal Density (CD), a measure proposed by Seth et al. to quantify the average Granger causality over a collection of time-series^25,26^; and 3) μ[ρ], a networks’ average Pearson correlation coefficient, a statistical measure of synchrony between the time-series of a network’s regions (see Fig. 1b). By including these measures in our analysis, we aim to emphasize the potential advantages to using μ[Φ^max^].

## Results

### Temporal and spatial control procedures

**Fig. 2:**
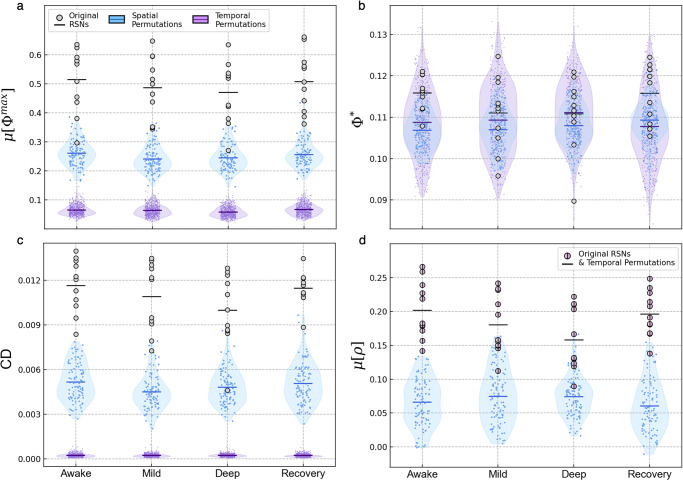
Results from spatial and temporal control procedures. For each of the four conditions, we generated 100 spatial control networks by randomly grouping five regions from different RSNs. Each RSN’s time-series was then randomly permuted 50 times in the temporal control procedure, leading to 550 randomized time-series for 11 RSNs in each condition. Note that these permutations and calculations were performed for time-series with all 17 subjects concatenated (see Methods for more details). We computed μ[Φ^max^] (**a**), Φ* (**b**), ${{{{{\rm{CD}}}}}}$ (**c**), and μ[ρ] (**d**) for the original RSNs (shown with the gray points) and both control permutations (shown with the violin plots). The horizontal lines corresponding to each distribution represent their mean. In the case of μ[ρ] (**d**), the overlapped data points indicate that the original RSNs’ values did not change when time points were permuted. For source data, see Supplementary Data 2. For statistical information, see Supplementary Note 6, Supplementary Table 3.

The two fundamental properties of each network are their spatial composition and time-dependent behavior, which depend on the cortical regions they include and the sequences of states appearing in their time-series, respectively. We hence started with a control procedure to evaluate the dependence of μ[Φ^max^] and the reference metrics on these properties. To test spatial dependency, we generated a series of control networks by randomly grouping five regions from different RSNs and computing μ[Φ^max^] for each random collection of regions. To evaluate temporal dependence, we maintained each network’s regions but permuted the sequences of states appearing in their time-series (i.e., the order of time-points was shuffled). In Fig. 2, we present the results of these permutations for each metric in comparison to the original networks.

For all conditions of awareness, almost all of the RSNs’ original μ[Φ^max^] values were significantly higher than those of the spatial control networks. This difference was even more drastic in the temporal control procedure. The networks behaved similarly when evaluated using CD. For both μ[Φ^max^] and CD, the default mode network (DMN) had the lowest values and was the only RSN not significantly different from the spatial control distributions (*p* > 0.05 for both measures in awake and deep sedation; see Supplementary Note 6 for statistical information). On the other hand, differences between the original networks and their permuted counterparts were insignificant for Φ*, which is clear from the large level of overlap between the plotted distributions. μ[ρ] dropped significantly for the spatial control networks, but this measure was completely unaffected by temporal permutations, which reflects the fact that ρ does not depend on the order of the sample used to compute it.

### Modulation patterns of μ[Φ^max^] during anesthesia

**Fig. 3:**
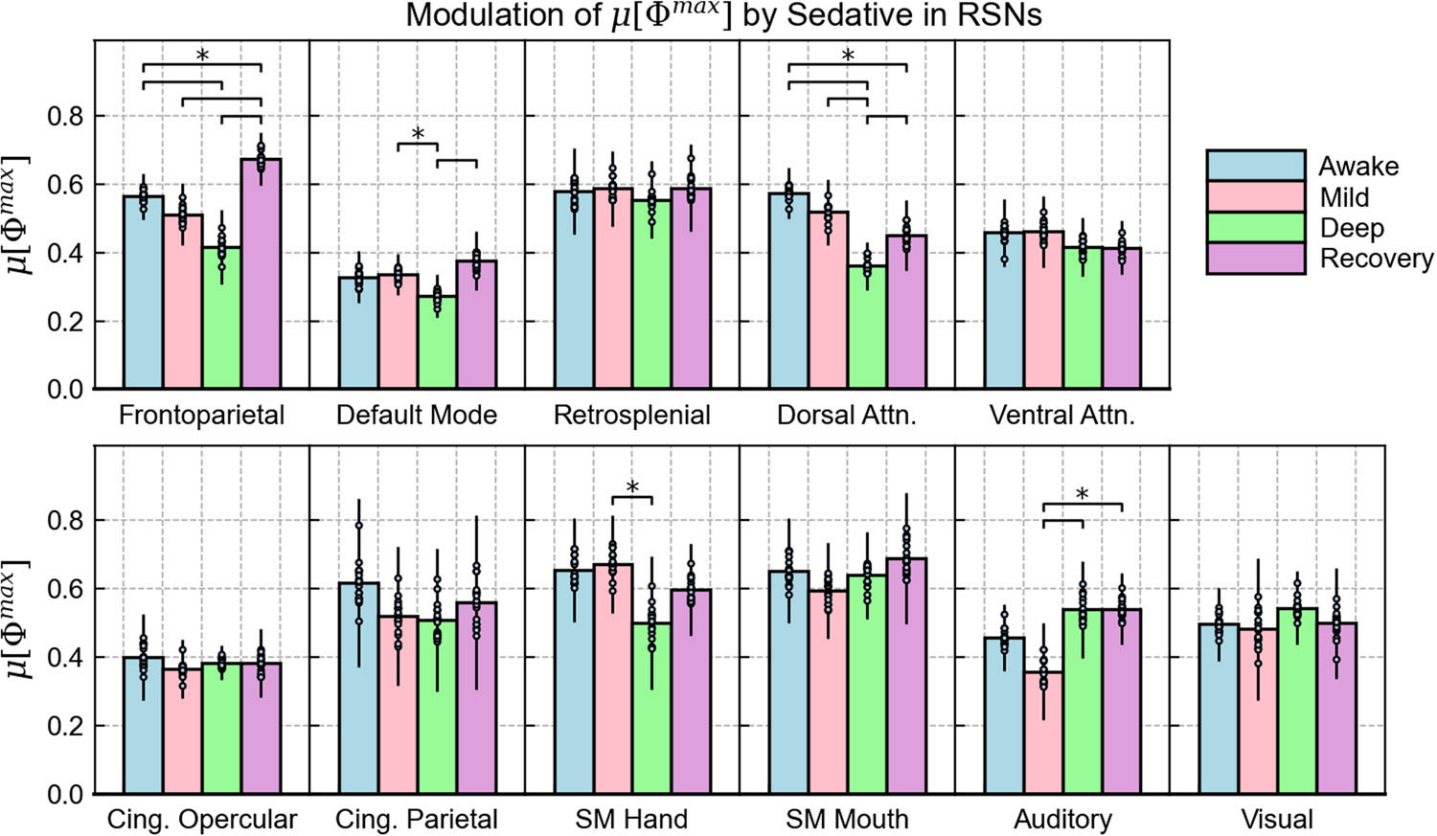
Modulation of μ[Φ^max^] by propofol in individual RSNs. For each network, the mean integrated information across the subject population is compared for the four conditions of awareness. Bar heights represent the mean of the sample of μ[Φ^max^] values, which was obtained by concatenating 16 subjects with a different subject left out for each measurement (*N* = 17), with individual data points given for each measurement. Error bars represent the population standard deviation, which was obtained by multiplying the standard error of the sample by √N. All statistically significant differences found between the conditions, such as awake vs. deep sedation, are indicated by the horizontal lines above the bars (*p < ${\alpha }^{{BH}}$, where ${\alpha }^{{BH}}$ is the significance threshold obtained with the Benjamini–Hochberg correction for multiple comparison, which varies when comparing different conditions. For all values derived from significance testing, see Supplementary Note 6; Supplementary Tables 4–7). For source data, see Supplementary Data 3.

Our central analysis focused on how propofol impacts the integrated information of specific RSNs. To account for possible variations across the population, we obtained a sample of 17 time-series for each network and condition. This was achieved by concatenating all but one of the subjects and excluding a different subject for each new time-series generated. The results for μ[Φ^max^] are shown in Fig. 3, where we present each network with a subplot that includes the distribution of values obtained for each condition. The RSNs are arranged according to their association with higher order cortical functions and conscious processing. All relevant statistical details (including correction for repeated comparisons over each network) are provided in the methodology as well as Supplementary Note 6; Supplementary Tables 4–7.

The networks that most clearly reflected subjects’ level of awareness were the frontoparietal (FPN) and dorsal attention networks (DAN). Both decreased gradually and reached a minimum in deep sedation with a statistically significant difference compared to the awake condition, which was followed by a significant increase in recovery. Interestingly, the FPN demonstrates an exceptionally large jump in this final transition, as its integrated information during recovery exceeds the value computed in the awake condition.

Like the FPN and DAN, the DMN’s μ[Φ^max^] value was lowest in deep sedation and its rebound in recovery presented a statistically significant increase. However, its overall modulation pattern was weaker, as no drop was observed between awake and mild sedation, and the drop in deep sedation was not statistically significant compared to the awake condition. Furthermore, the DMN yielded the lowest μ[Φ^max^] values compared to all other networks.

Other RSNs demonstrated minor and statistically insignificant fluctuations across the four conditions. Out of the higher-order networks, these included the retrosplenial, ventral-attention, and cingulate networks. Turning to the sensory cortices, the auditory network demonstrated a stronger degree of fluctuation with a substantial drop in mild sedation. However, this modulation pattern was inconsistent with changes to subjects’ conscious state. While the SM hand network presents a significant difference between mild and deep sedation, the SM mouth and visual networks did not demonstrate any significant variations in μ[Φ^max^].

### Modulation patterns for the reference metrics

**Fig. 4:**
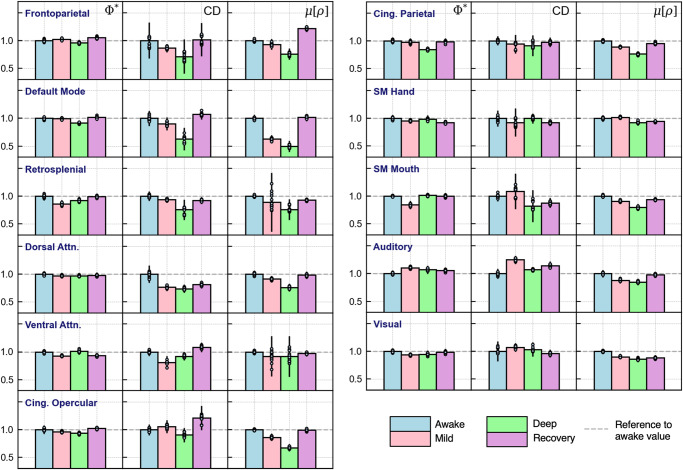
Modulation of reference metrics by propofol in individual RSNs. The networks, presented across rows, are analyzed using the reference metrics Φ*, CD, μ[ρ], which were assigned to columns. As before, each network is analyzed over the four conditions of awareness. To focus on each network’s behavior under anesthesia, the values were normalized with respect to each network’s awake value (set to equal 1). Bar heights represent the normalized means of CD, Φ*, and μ[ρ] values obtained by concatenating 16 subjects with a different subject left out for each measurement (i.e., these measures were applied to the same set of time-series used to compute μ[Φ^max^] in Fig. 3). Error bars represent the population standard deviation, which was obtained by multiplying the standard error of the sample by √N. For absolute measurements and statistical tests, see Supplementary Note 3; Supplementary Figs. 2–4, which includes an equivalent version of Fig. 3 for each measure. For source data, see Supplementary Data 3.

To see how the networks behaved with respect to each reference metric, we repeated the previous analysis for Φ*, CD, and μ[ρ], which we present in Fig. 4. Since the measures varied in magnitude, we normalized the values of the four conditions with respect to each network’s awake value. For absolute values and statistical information, an equivalent version of Fig. 3 is included for each metric in Supplementary Note 3; Supplementary Figs. 2–4.

Out of all measures, Φ* presented the lowest level of variability with respect to changes in awareness level. This is clear from the first column of Fig. 4, where most networks demonstrate relatively minor fluctuations throughout sedation. One similarity between Φ* and μ[Φ^max^] was the behavior of the DMN, which also showed negligible change in mild sedation but a significant drop in deep sedation and a rebound in recovery. Whereas μ[Φ^max^] was strongly modulated in the FPN and DAN, Φ* closely corresponded to awareness level in the two cingulate networks.

On the other hand, the behavior of CD was more similar to that of μ[Φ^max^]; not only were there heterogeneities among the networks’ modulation patterns, but there was significantly more variability in this metric’s values. As with μ[Φ^max^], the FPN and DAN accurately reflected changes to subjects’ level of awareness, while the sensorimotor, auditory, and visual networks presented no clear sedative-induced changes. However, CD also mirrored the conscious evolution of subjects in the DMN and retrosplenial network, which was not the case for μ[Φ^max^].

Finally, μ[ρ] also showed substantial changes across different conscious states, suggesting that this measure is strongly impacted by anesthesia. However, we emphasize that with correlation, nearly all networks demonstrate a modulation pattern that corresponds to the conscious evolution of subjects. The only exception was the SM Hand network, which increased moving from awake to mild sedation. Otherwise, μ[ρ] demonstrates a more globalized effect across the networks compared to the other metrics.

### Modulation and magnitude

**Fig. 5:**
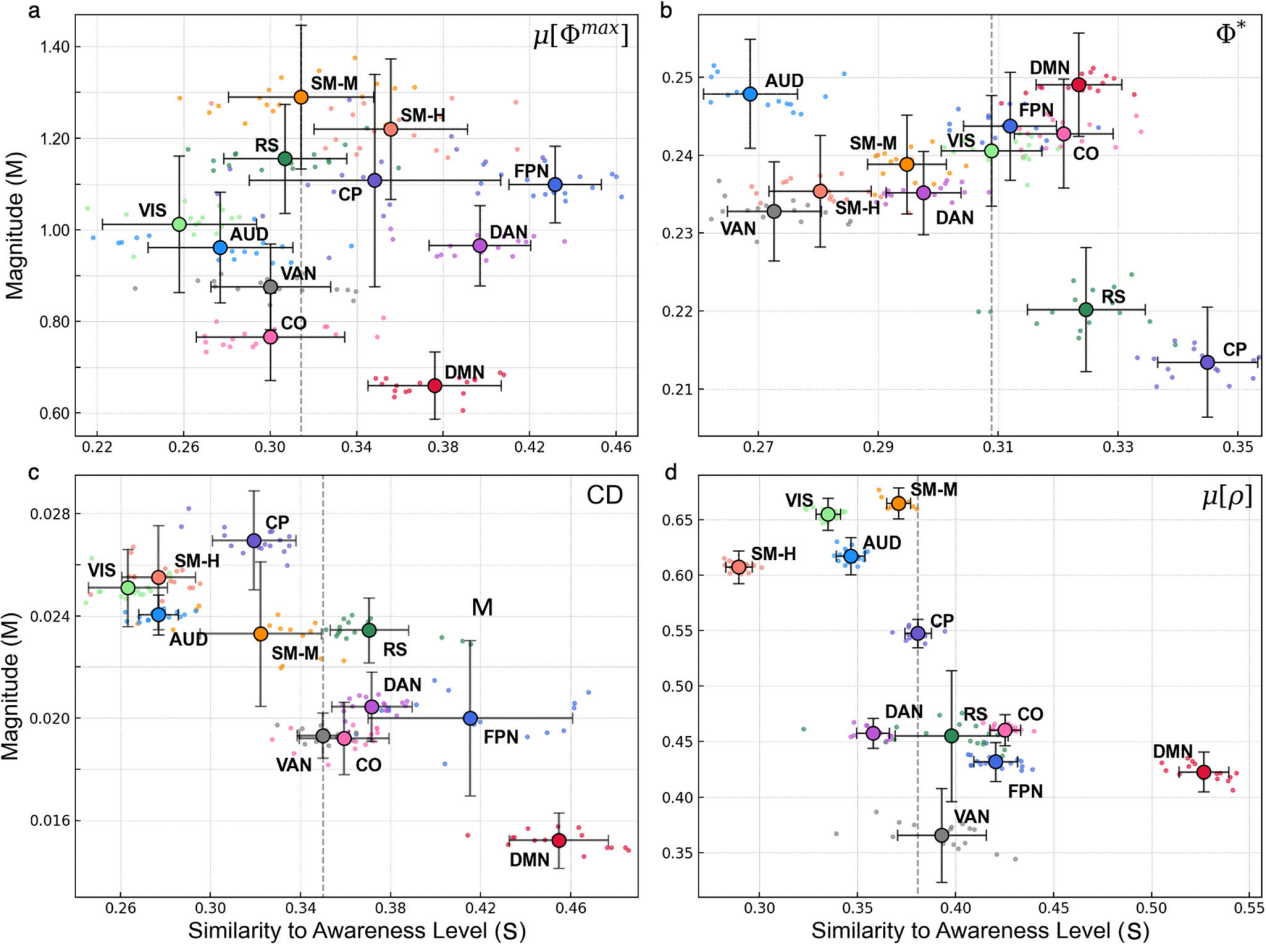
Magnitude (M) vs. degree of modulation pattern that reflects awareness level (S). The overall magnitude (M) generated for a particular RSN is plotted against the degree to which a network reflects the four conditions of awareness (S) for each metric of interest: μ[Φ^max^] (**a**), Φ* (**b**), CD (**c**), and μ[ρ] (**d**). Vertical error bars represent the standard deviation of each network’s M, while horizontal error bars represent the standard error of each network’s S value. Within each subplot, the dashed vertical lines correspond to the median value of S obtained out of all networks. We use this median to separate RSNs whose modulation patterns strongly correspond to changes in awareness level. Networks are labeled as: FPN frontoparietal, DMN Default mode, RS Retrosplenial, DAN Dorsal attention, VAN Ventral attention, CO Cing. Opercular, CP Cing. Parietal, SMH Sensorimotor hand, SMM Sensorimotor mouth, VIS Visual, AUD Auditory. For source data, see Supplementary Data 4.

The results presented so far suggest two ways to interpret an RSN’s integrated information. The first, which we already discussed qualitatively, is a network’s modulation pattern and the degree to which it reflects changes in subjects’ awareness level. The second is the overall magnitude of integrated information within the network. To analyze these aspects quantitatively, each network’s four μ[Φ^max^] values were arranged into a four-element vector in the appropriate order (i.e. [μ[Φ^max^]_A_, μ[Φ^max^]_M_, μ[Φ^max^]_D_, μ[Φ^max^]_R_]). This vector’s magnitude was used to quantify the overall amount of integrated information generated by a certain network (M). We then obtained a measure quantifying the degree to which a network’s modulation pattern corresponds to changes in awareness level (S). This effectively represents the extent to which μ[Φ^max^] drops moving towards deep sedation and increases in recovery (see Methods for more details). We computed M and S values for each network in terms of μ[Φ^max^] and the reference metrics, which are presented in Fig. 5.

While these results reinforce our previous descriptions of RSNs and their modulation patterns, they also provide insight into how the networks behave with respect to one another. First, it is important to note that while some networks may accurately reflect changes to conscious level, they do not necessarily yield a high level of integrated information. In the case of μ[Φ^max^], the FPN and DAN had the highest values of S. However, the highest value of M was computed for the SM Mouth network, where changes to μ[Φ^max^] did not closely reflect the conscious evolution of subjects. The DMN also had higher S compared to most other networks, but its overall magnitude of μ[Φ^max^] was the lowest.

A similar argument could be made for the reference metrics. In the case of CD, the DMN had the lowest magnitude while demonstrating the largest value of S. The same could be said for the cingulate-opercular network in terms of Φ*, while for μ[ρ], the four networks with the highest magnitudes were all below the median S value. Moreover, the networks had the greatest median value of S for μ[ρ], which reflects our previous observation of a more globalized effect through this metric.

Finally, these results also emphasize differences in magnitude variability across the metrics. Taking all the networks’ M values into account, the metric with the highest variability (computed as the percent difference between the maximum and minimum values) was μ[Φ^max^] (62%), meaning there was a sizable difference between networks that generated the most integrated information (i.e., SM networks) and those that generated the least (i.e., DMN). The networks also demonstrated substantial variability through CD and μ[ρ] (53 and 58%, respectively), while Φ* had the lowest variability (15%), suggesting that it is less sensitive to inter-network differences.

## Discussion

Although much knowledge has been gained through the use of advanced neuroimaging techniques, consciousness continues to be a highly debated topic.^2,9^ Of the many attempts made to explain conscious phenomena, Integrated Information Theory (IIT) has emerged as one of the leading perspectives. Starting with phenomenology, IIT defines the qualitative aspects of a conscious experience and introduces a measure known as integrated information (Φ) to quantify the degree to which a system is concious.^10,12,15^

While the ideas of this framework are promising, extracting an empirical measure of Φ has proven to be challenging. Some progress has been made to implement measures of Φ to neuroimaging data^18,19^, though existing literature remains mostly theoretical and speculative. Furthermore, there exist no empirical applications of the latest and most advanced formulation of IIT (IIT 3.0), which introduces a thorough measure of conscious level called the maximally integrated conceptual information, or Φ^max^.

While there are several limitations to extracting this measure from neuroimaging data, this paper introduced an implementation of IIT 3.0 to resting-state fMRI. Starting with data acquired from subjects who underwent propofol-induced anesthesia, we analyzed sedative-induced changes in integrated information over 11 resting-state networks (RSNs). The metric extracted using IIT 3.0 was μ[Φ^max^], or the weighted average of Φ^max^ over a network’s time-series. As reference points to our results, we included three additional metrics that were previously used as potential indicators of conscious level: 1) Φ*, a measure of integrated information derived from IIT 2.0, 2) Causal Density (CD), a network’s average Granger causality, and 3) μ[ρ], a network’s average Pearson correlation coefficient.

Our first analysis included two control procedures where we applied random permutations to the spatial and temporal arrangements of the networks. In both controls, μ[Φ^max^] dropped significantly, and the drop was especially drastic following temporal permutations. This is an important finding because it indicates that the RSNs of interest generate an intrinsic level of integrated information that vanishes when their spatial and temporal properties are disrupted. Similar results were obtained with CD, meaning that Granger causality could also capture the spatial and temporal characteristics of the networks in question. In Supplementary Note 7 (Supplementary Figs. 6–9), we present a more detailed analysis of temporal permutations and their effects on μ[Φ^max^] and CD, which provides important insight into the causal properties measured by both metrics.

At the same time, Φ* did not demonstrate a strong dependence on these properties, as its values did not drop significantly in these procedures. The failure to differentiate a network’s original time-series from one that is randomized means that unlike μ[Φ^max^] or CD, Φ* did not effectively capture the BOLD signal’s causal relationships and is hence less robust in analyzing a network’s time-dependent behavior. These results may reflect the fact that Φ* is computed using mutual information, which is an entropic measure. Mutual information is defined by the entropy overlap of two probability mass functions, which in the case of Φ*, represent the probabilities of the system’s current and past states.^13,16,17^ Therefore, the insignificant change of this measure may indicate that permutations had limited impact on these probabilities, as they changed the order but not the overall content of states appearing in a time-series.

When analyzed using Pearson correlations, the random grouping of cortical regions significantly decreased μ[ρ] as compared to the original RSNs. On the other hand, temporal permutations had no effect on this measure. These results are not surprising, as the networks were constructed using correlational functional connectivities in the first place, and the Pearson correlation coefficient does not depend on the order of the data used to compute it. While correlations are fundamental in fMRI analysis, their insensitivity to temporal permutations emphasize the need to implement additional metrics when analyzing the BOLD signal’s time-dependent and causal properties.^27^

Our central investigation focused on changes to μ[Φ^max^] throughout sedation. In general, the effects of anesthesia differed among the RSNs. The modulation pattern that corresponds to the conscious evolution of subjects (i.e., a decrease moving from wakefulness to deep sedation followed by an increase in recovery) was most clearly observed in the frontoparietal and dorsal attention networks (FPN and DAN, respectively).

The FPN, also known as the central executive network, is a crucial hub for cognitive control and goal-oriented behavior.^28–30^ It is said to connect external stimuli with stored internal representations, and hence plays an important role in integrating inputs from several cortical regions.^31^ Accordingly, its drop in μ[Φ^max^] coincides with the loss of these functions as subjects become deeply sedated. This network’s behavior is further explained by findings that propofol inhibits functional connections between the frontal and parietal cortices, which form the basis to this RSN.^23^ Interestingly, the FPN’s integrated information peaked in the recovery stage, which could indicate a state of hyperexcitability that results in a broad integration of inputs during the physiological rebound of consciousness.

The DAN demonstrated a similar behavior to the FPN, albeit without a drastic increase in recovery. This network facilitates top-down, voluntary mediation of external attention that is primarily associated with visuospatial orienting.^32,33^ Considering the related functions and noted interactions between the FPN and DAN^30^, their similar behaviors are a promising finding. On the other hand, several networks including the ventral attention, retrosplenial, and cingulate networks demonstrated minor and statistically insignificant fluctuations of μ[Φ^max^]. Although some significant changes were demonstrated by the sensory RSNs, we did not find any consistent relationship between their integrated information and the condition of subjects.

Another important network to discuss is the DMN, which presented the lowest μ[Φ^max^] values compared to all other networks. This may indicate that despite a high metabolic activity at rest^34^, the DMN’s integrated information as an isolated system is intrinsically low. Perhaps, an analysis of its interactions with other cortical regions is necessary to better understand its integrative mechanisms.^35,36^ This would be in line with the results of the spatial control procedure, where the DMN’s μ[Φ^max^] values were comparable to those of the control networks (i.e., randomly grouped cortical regions), which themselves contain interactions between regions of the DMN and those of other RSNs.

Overall, our results for μ[Φ^max^] support previous findings that propofol preferentially suppresses higher-order networks with regions in the frontal and prefrontal cortices, which are commonly attributed to conscious awareness.^22,37,38^ At the same time, it was also found that propofol’s effects may be weaker for sensory cortices, which may explain the lack of modulation for these RSNs.^37,39^

To solve the problem of high computational cost in IIT 3.0, one proposed solution is to estimate integrated information at the mechanism level.^40,41^ While the full calculation of Φ^max^ involves computing the integration of all mechanisms with respect to one another, this alternative approach only considers the integrated information of individual mechanisms. In Supplementary Note 4 (Supplementary Fig. 5), we apply this method and compare it to our results with μ[Φ^max^]. In summary, several networks behave differently when evaluated with mechanism-level integrated information, suggesting that the rigorous yet complete calculation of Φ^max^ may not always be predicted by analyzing mechanisms individually.

While our central analysis focused on μ[Φ^max^], we further evaluated each network’s time-series using the reference metrics (see Table 1 for a brief summary of these results). First, CD demonstrated notable similarities to μ[Φ^max^]; it reflected changes to conscious level in the FPN and DAN, while its values in the sensorimotor, auditory, and visual networks demonstrated no significant modulation. On the other hand, the DMN and retrosplenial network reflected changes to awareness level more closely than they did for μ[Φ^max^].

**Table 1:** Summary of results comparing μ[Φ^max^] and the reference metrics.

	μ[Φ^max^]	CD	Φ*	μ[ρ]
Spatial control	Significant drop	Significant drop	Change, but no significant drop	Significant drop
Temporal control	Significant drop: strong dependence on order of points in time-series	Significant drop: strong dependence on order of points in time-series	No significant drop: weak dependence on order of points in time-series	No change: no dependence on order of points in time-series
RSNs reflecting awareness level	FPN, DAN	FPN, DAN, DMN, Retrosplenial	Cingulate networks	All except SM Hand

The results of the control procedure and the analysis of modulation in each measure are briefly summarized above. Different aspects of our analysis are given across rows, and the four metrics are presented in the columns.

Turning our attention to Φ*, the cingulate networks were the only ones to demonstrate a steady decrease moving towards deep sedation and an increase in recovery, while other networks typically associated with awareness, such as the FPN and DAN, did not demonstrate a meaningful modulation pattern. The only network that behaved in similar fashion to μ[Φ^max^] was the DMN, which dropped during deep sedation but demonstrated a negligible difference between awake and mild sedation. Compared to other metrics, the magnitude of Φ* had much lower variability across different networks, meaning it could not distinguish RSNs with high and low integration as effectively as μ[Φ^max^].

While μ[Φ^max^], CD, and Φ* exhibited a variety of sedative-induced changes across different networks, this was not the case for μ[ρ]. When analyzed using the Pearson correlation coefficient, nearly all networks demonstrated a modulation pattern corresponding to the conscious evolution of subjects. On the surface, this may appear to indicate that μ[ρ] is a more robust measure of conscious level. However, this would contradict the well-established principle that certain cortical regions are more important to conscious processing than others. Indeed, the global behavior of μ[ρ] would make it difficult to determine which networks are responsible for consciousness and higher order cognitive functions. Perhaps, the homogenous behavior of μ[ρ] reflects a global neurophysiological effect that is induced by the sedative.^39,42^ Moreover, previous studies found that various physiological changes, such as eye opening or closing, heart rate, and respiration can significantly influence measures of functional connectivity,^43,44^ but these do not necessarily correspond to increased or reduced levels of awareness.

Taken together, the issues pointed out for μ[ρ] and Φ* suggest that μ[Φ^max^] and CD are better candidates for an effective measure of conscious level; both were sensitive to the BOLD signal’s time-dependent behavior, both reflected changes to awareness in networks typically associated with consciousness, and both exhibited heterogeneity in their behaviors across different cortical regions. What remains to be addressed is how the two metrics differ from one another. While the relationship between cause and effect is essential to both, CD is limited to measuring a network’s causal interactions. On the other hand, Φ^max^ extends on causation and quantifies the extent to which this property is integrated. According to IIT, integration is essential to consciousness, and computing it makes Φ^max^ a considerably more sophisticated measure than CD.

Although Granger causality is a useful tool with many potential biological applications, there are theoretical problems with using a measure of conscious level that relies exclusively on causality. The nervous system contains a multitude of causal relationships that do not necessarily contribute to consciousness, such as sensory pathways and interactions within the autonomic nervous system.^9,12^ Even within the brain, there are sophisticated structures consisting of complex neuronal interactions that are not attributed to consciousness (i.e., the cerebellum). IIT argues that these components of the nervous system lack integration, whereas highly integrated structures such as the cerebral cortex do generate consciousness.^45^

Therefore, the differences in our results for μ[Φ^max^] and CD can be attributed to whether or not integration was computed. While the DMN and retrosplenial network showed significant changes through CD, they did not do so through μ[Φ^max^], meaning that only the FPN and DAN reflected changes to awareness level through both causality and integration. Even if μ[Φ^max^] is a more computationally intensive metric, the inclusion of integration renders it a stronger measure of properties associated with consciousness.

In our final analysis, we considered two features derived from the four conditions of awareness, which were a network’s overall magnitude of integrated information and the degree to which its evolution reflects changes to awareness. The results indicated that while some cortical networks generate a high level of integrated information, their modulation patterns do not reflect the effects of the anesthetic. In our case, the sensorimotor networks generated the highest μ[Φ^max^] with relatively weak sensitivity to sedation. Although the reference metrics placed emphasis on different networks, they also demonstrated a lack of parallelism between high magnitude and reflection of awareness level.

These results are important in the context of ongoing debate about which cortical regions possess the capacity for consciousness. Recent theories challenge the emphasis previously placed on the FPN and instead attribute consciousness to a posterior cortical hot zone, which spans occipital, temporal, and parietal regions.^9,46^ While this idea is not supported by our results, further research is needed to determine the extent to which these regions contribute to conscious processing. Nevertheless, our findings emphasize the importance of analyzing integrated information in terms of both modulation and magnitude. The possibility of cortical regions being highly integrated but weakly modulated is an important factor to consider when implementing measures of conscious level such as Φ^max^.

While our investigation yielded promising findings, it is important to discuss several limitations to our procedure and implementation. First, a disadvantage of fMRI is its low temporal resolution, which ranges between 2 to 3 s for consecutive time points.^47^ Computing causality for fMRI data is hence controversial, as its low sampling rate may preclude a meaningful evaluation of causal properties.^25^ While there is evidence to support a neuronal basis for the BOLD signal, fMRI measures a hemodynamic response rather than neuronal activity, which occurs at a much faster timescale and is absolutely crucial for understanding consciousness. Another issue of studying anesthesia with fMRI is the increased likelihood of head motion during sedation, which can significantly affect image quality.^48,49^

In terms of our computational scheme, the analysis of time-series was constrained by the Markovian assumptions underlying IIT 3.0.^21^ We tested for these requirements and found that the fMRI time-series did not satisfy the conditional independence property, meaning that some information in the original signals was lost when inputted to PyPhi (see Supplementary Note 5; Supplementary Tables 1, 2 for results on the Markov property and conditional independence tests). Another limitation is the high computational cost of Φ^max^ and how it scales with network size. Although an improved spatial resolution may provide a better perspective for analyzing an RSN’s causal dynamics, the computational scalability of *O(n53*^*n*^*)* means that a sixth region would increase calculation time by over sixty-fold.

Furthermore, our procedure consisted of concatenating time-series from different subjects to obtain longer signals for each RSN and condition. While this was done to address the issues of using short time-series, inter-subject differences are inevitable, and as a result, the concatenated signals contained discontinuities that would not have existed if longer signals were obtained from individual subjects. PyPhi also requires systems whose elements take on binary states, which allows for no consideration of the BOLD signal’s continuous nature.

Nevertheless, our approach to calculating integrated information from empirical fMRI data produced meaningful results that merit further investigation of this framework. For future implementations, we suggest a procedure that computes Φ^max^ for the time-series of individual subjects, which will require the acquisition of signals with more time points than used in this analysis. Although our study focused on anesthesia with propofol, there are other conditions in which Φ^max^ should be investigated. For instance, our proposed procedure could be applied in an fMRI study that compares healthy controls to patients suffering from disorders of consciousness.^5,50^ These pathologies have become a subject of growing concern in neuroscience, and IIT may provide useful insight about them.^2,45^

Although our study focused on integrated information theory, further research should incorporate other theories that have been proposed to explain consciousness. IIT continues to be debated, as the validity of its axioms on conscious phenomenology has been challenged.^51,52^ Another widely discussed framework is global workspace theory (GWT), which attributes conscious percepts to spotlights of attention that are mediated by executive control functions.^53,54^ GWT and IIT differ in several ways; one critical distinction is that GWT attributes consciousness to whole-brain interactions between different networks, whereas IIT focuses on how consciousness can arise due to a network’s intrinsic causal properties.

While these frameworks present competing ideas, recent efforts have been made to study them in parallel.^55,56^ Such developments are a promising step, as certain aspects of consciousness may be better understood with one theory over another. Incorporating several perspectives on consciousness could also allow for an approach in which differing frameworks complement, rather than contradict, one another. In conclusion, the multifaceted nature of consciousness calls for a multifaceted approach to advance our understanding of this astonishingly sophisticated concept.

## Methods

### Participants and ethics

We recruited 17 healthy volunteers (4 women; mean age 24 years, SD = 5) after posting printed advertisements throughout the university and sharing the study through word of mouth. All were native English speakers, right-handed, and had no history of neurological disorders. The attending MR technician and anesthesiologist instructed volunteers to complete safety screening questionnaires for MRI and propofol, which was followed by written informed consent forms to confirm their understanding of any potential risks involved. We remunerated volunteers for their time and participation. Ethical approval was obtained from the Health Sciences Research Ethics Board and Psychology Research Ethics Board of Western University (REB #104755).^57^

### Administration of propofol

In preparation for sedation, a 20 G i.v. cannula was inserted into a vein in the dorsum of the non-dominant hand and a propofol infusion system was connected to it. Intravenous propofol was administered with a Baxter AS 50 syringe pump (Singapore). To deliver propofol in an incremental, stepwise fashion, an effect-site/plasma steering algorithm was used in combination with a computer-controlled infusion pump. The infusion pump was adjusted to achieve the desired level of sedation, which was guided by target propofol concentrations predicted by the TIVATrainer (the European Society for Intravenous Anesthesia, eurosiva.eu) pharmacokinetic simulation program. This model provided target-controlled infusion by adjusting propofol infusion rates, with the goal of reaching and maintaining the target blood concentrations specified by the Marsh 3 compartment algorithm for each participant (also incorporated in the TIVATrainer software).^58^ Subjects underwent four conditions throughout sedation and acquisition; Awake: Propofol was not yet administered. Participants were fully awake, alert, and communicative; Mild sedation: At the start of this phase, we began propofol infusion with a target effect-site concentration of 0.6 µg/ml. Oxygen was titrated to maintain SpO_2_ above 96%. After reaching the target effect-site concentration, we assessed the participants’ level of sedation and maintained the effect-size concentration if observations were consistent with mild sedation. Initially, participants became calmer and slow in their responses to verbal communication. Once they stopped engaging in spontaneous conversation, became sluggish in speech, and only responded to loud commands, they were classified as level 3 using the Ramsay sedation scale^59^ and considered mildly sedated; Deep sedation: Prior to reaching the deep sedation phase, the target effect-site concentration was increased in increments of 0.3 µg/ml and responsiveness was assessed with each increase. Once participants reached level 5 on the Ramsay scale of sedation, whereby they stopped responding to verbal commands and were unable to engage in conversation, the level of propofol was maintained. Participants remained capable of spontaneous cardiovascular function and ventilation; Recovery: Propofol administration was terminated after acquisition in deep sedation. Approximately 11 min afterwards, participants reached level 2 on the Ramsey scale, which was marked by clear and quick responses to verbal commands.

The mean estimated effect-site propofol concentration was 2.48 (1.82–3.14) µg/ml, and the mean estimated plasma propofol concentration was 2.68 (1.92–3.44) µg/ml. The mean total mass of propofol administered was 486.58 (373.30–599.86) mg. The variability of these concentrations and doses is typical for studies of the pharmacokinetics and pharmacodynamics of propofol.^60,61^

Prior to initiating fMRI acquisition, three independent assessors (two anesthesiologists and one anesthesia nurse) evaluated participants with the Ramsay scale. Participants were also asked to perform a basic verbal recall memory test and a computerized (4 min) auditory target detection task, which further assessed each participant’s wakefulness/sedation level independently of the anesthesia team. Scanning commenced only after agreement on the wakefulness/sedation level among the three anesthesia assessors.

### Neuroimaging data preprocessing

Echo-planar sequencing was used to acquire functional images with the following properties: 33 slices, voxel size: 3 × 3 × 3 mm^3^, inter-slice gap of 25%, TR = 2000 ms, TE = 30 ms, matrix size = 64 × 64, FA = 75°. Resting-state scans had 256 vol. We also obtained an anatomical scan using a T1-weighted 3D MPRAGE sequence with the following properties: 32 channel coil, voxel size: 1 × 1× 1 mm^3^, TE = 4.25 ms, matrix size = 240 × 256 × 192, FA = 9°.

T1 images were preprocessed using the following toolboxes: SPM (http:www.fil.ion.ucl.ac.uk/spm), FSL (https://fsl.fmrib.ox.ac.uk/fsl/fslwiki/), SimpleITK (http://www.simpleitk.org/) and Dipy (http://nipy.org/dipy/).

Preprocessing for T1-weighted imaging consisted of the following: manual removal of the neck, removal of non-brain tissue using the FMRIB Software Library (FSL), correction of non-uniformity in low frequency intensity based on the N4 bias field correction algorithm (obtained from SimpleITK), image denoising with the nonlocal means algorithm from Dipy, and spatial normalization to standard stereotactic Montreal Neurological Institute (MNI) space using the SPM12 normalization algorithm. The three initial volumes were discarded to avoid T1 saturation effects in the fMRI data. Head motion and slice timing correction were performed using the MCFLIRT algorithm from FSL. Further artifact correction was performed using RapidArt (https://www.nitrc.org/projects/rapidart/), a software that employs an outlier-based algorithm to detect head motion exceeding 3 mm in the brain signal. A rigid body transformation, which was obtained from head-motion correction with FSL, yielded six motion parameters for translation and rotation in three dimensions. The time-series were further cleaned by removing spurious variance with nuisance signal regression, which was based on the average time series of external regions of noninterest (white matter and cerebrospinal fluid).^62^ fMRI data were subsequently co-registered onto the T1 image and spatially normalized to the MNI space with the SPM12 normalization algorithm. Finally, spatial smoothing was applied to the fMRI data using a Gaussian kernel (8 mm full width at half maximum as implemented in SPM12).

### Extraction of representative regions for RSNs

**Fig. 6:**
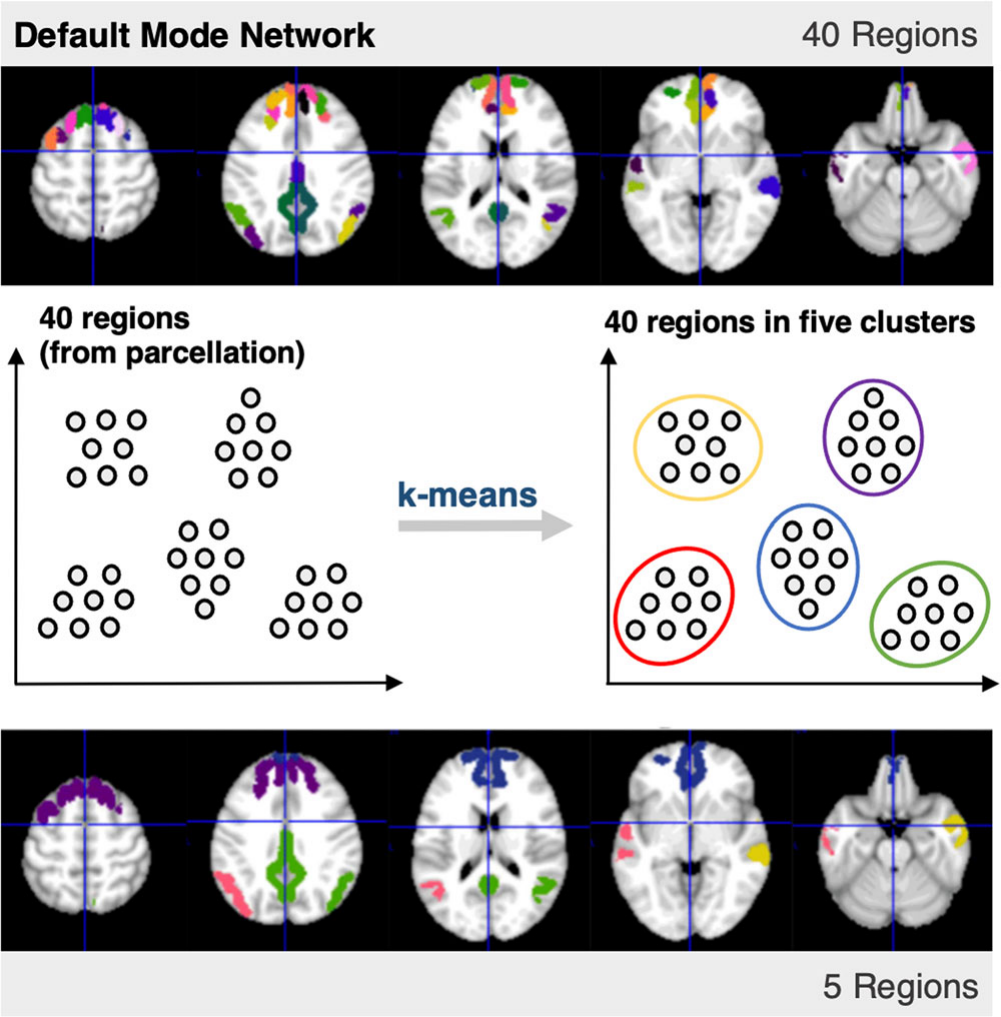
Obtaining five representative regions for an RSN. Starting with 40 regions in the DMN, a k-means algorithm was used to obtain five clusters, each associated with a centroid. The regions originally associated with each RSN were narrowed down to five clusters, which yielded the five regions used in our analysis. For the representative regions included in each of the 11 RSNs, see Supplementary Note 1; Supplementary Fig. 1.

A resting-state cortical parcellation scheme (presented by Gordan et al., 2016) was applied to the images.^63^ The resting-state networks (RSNs) extracted were the auditory, retrosplenial, ventral, visual, cingulo-opercular, cingulo-parietal, default mode, dorsal, sensorimotor hand, and sensorimotor mouth networks. The original list of parcels and the networks they correspond to can be found on the website of the Petersen Neuroimaging Lab, Washington School of Medicine in St. Louis (https://sites.wustl.edu/petersenschlaggarlab/parcels-19cwpgu/). The cortical areas were grouped into representative regions of interest (ROI), which were selected from five clusters by running a k-means algorithm over the spatial centroids of each RSN. Five regions were included in each extracted network to maintain reasonable balance between spatial resolution and computational complexity. A summary of this procedure is presented in Fig. 6.

### Obtaining standardized and binarized time-series

Once the five representative regions of each network were obtained, their means were obtained. The time-series were then detrended and filtered using a bandpass Butterworth filter with cut-off frequencies set at 0.01 Hz and 0.1 Hz. We then standardized each time-series with respect to its own mean and standard-deviation. Regions with a positive z-score for a particular time point were set to 1 (above-baseline activity), and those with a negative z-score were set to 0 (below-baseline activity). This allowed for an RSN to be in any one of 32 (2 ^*N=5*^) possible states at a certain time point. For example, the state [1, 1, 1, 1, 1] represents above-mean activity in all the regions of an RSN. The time-series array for each RSN, subject, and condition had dimensions of 245 × 5 (number of time points × number of regions).

### Obtaining the transition probability matrix

The software used to implement IIT was PyPhi, a publicly available Python module recently developed in accordance with IIT 3.0 (https://pyphi.readthedocs.io/en/latest/).^21^ When calculating Φ^max^, the principal input is the Transition Probability Matrix (TPM), which describes the mechanisms governing a system’s behavior through probabilities of transitioning between states. There are two variants of the TPM in PyPhi: 1) the state-by-state TPM describes the probability that any given state of the entire RSN transitions to another state in a subsequent time point, with dimensions: *N*_*states*_ × *N*_*states*_ (32 × 32), and 2) the state-by-node TPM describes the probability of a node flipping from (0 to 1 or 1 to 0) when the system is in a certain state, with dimensions *N*_*states*_ × *N*_*nodes*_ (32 $\times$ 5).

First, a state-by-state transition probability matrix was generated directly from the time series. This was accomplished by counting the number of times each state transitioned to any other state. States were assigned an index following little-endian (LE) convention, and the count of transitions was used to populate a 32 × 32 square-matrix. To normalize the matrix, each row was divided by the number of times the state corresponding to its index appeared in the time series and transitioned to another state. For example, if 5 transitions occurred from the state [1, 0, 0, 0, 0] (LE index = 1) to the state [1, 1, 0, 0, 0] (LE index = 3), the entry in row 1 and column 3 was set to equal 5. If 20 transitioned occurred from the first state to any other state in the time-series, the row was then normalized to give a value of 0.25 for this entry (i.e., 25% probability for the system to transition to state 3 when in state 1).

The time series for each subject and condition consisted of 245 time points, which was a relatively small number considering the 32 possible states for each network. This was problematic as it yielded sparse transition probability matrices with significant inter-subject variation, resulting in spurious calculations of Φ^max^. This problem was addressed by concatenating (linking) the time-series of several subjects. This resulted in longer time series, which we hoped would provide sufficiently populated TPMs. While there are limitations to this approach, we hoped that concatenating would produce TPMs where the predominant mechanisms contributing to Φ are those likely to be found across several subjects and are hence intrinsic to a particular network.

### Calculating average integrated information μ[Φ^max^]

To calculate Φ^max^, the TPM of the concatenated signals was converted to a state-by-node form and then inputted to PyPhi to generate a network class. Φ is calculated for a particular subsystem *S* of the network class, which, along with the TPM that specifies network’s mechanisms, comprises of 1) the state of the system at a given time, which sets the necessary background conditions, and 2) the subset of nodes to be included in the subsystem, which is used for irreducibility analysis.

**Fig. 7:**
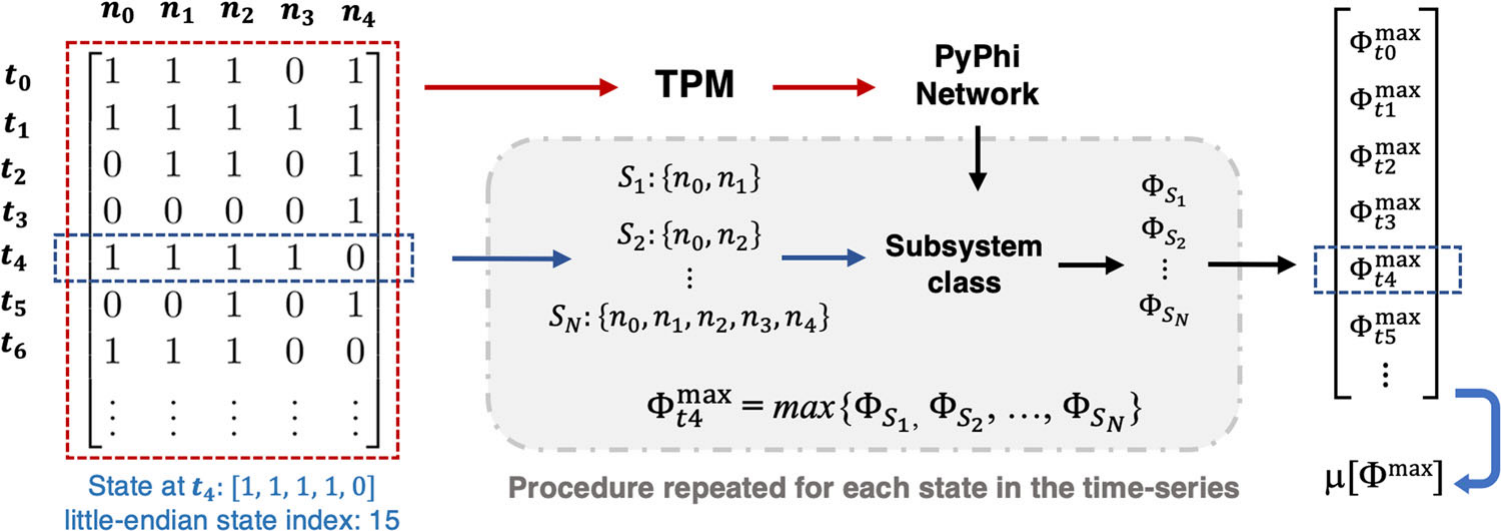
Summary of procedure for computing μ[Φ^max^]. Each binarized time-series was used to construct a transition probability matrix (TPM) with dimensions 32 × 32, which was constructed by counting the number of times any one state transitioned to any other. Calculations of Φ are performed for a subsystem, which is defined by the state of the system and a subset of two or more nodes within it. For each state appearing in the time-series, the maximally integrated conceptual information Φ^max^ was obtained from the subset of nodes that maximizes Φ. Finally, we count the occurrences of each state and compute the weighted average of Φ^max^ over the time-series, which we refer to as μ[Φ^max^].

Typically, Φ is computed for every possible subset of a network’s regions, and the system’s Φ^max^ (maximally integrated conceptual information) at a certain state is defined as the maximum value obtained from the values of all subsets. As the state of the networks (and hence Φ) varies throughout its time series, we calculated Φ^max^ for every state and obtained a weighted average, which we denote as μ[Φ^max^]. The contribution (weight) of each state was based on the frequency of its occurrence in the time-series. We found that the subset yielding Φ^max^ in each RSN and condition was the one including all five regions. For computational efficiency, it was therefore possible to evaluate only the full-network subset. Each step needed to obtain μ[Φ^max^], starting with the time-series and the subset analysis, is summarized in Fig. 7.

### Calculating causal density

To compute Causal Density (CD), we implemented the Multivariate Granger Causality Toolbox (https://www.mathworks.com/matlabcentral/fileexchange/78727-the-multivariate-granger-causality-mvgc-toolbox).^26^ For time-series data, Granger causality is obtained using a vector auto-regressive (VAR) model of order *p*. Each time Granger causality was calculated, an appropriate value of *p* was determined by testing values of *p* between 1 and 20; a range chosen to balance quality of fit and the potential for overfitting. For each network’s binarized time-series, we computed the bidirectional Granger causality for every pair of regions and obtained CD by averaging over the values of all pairs.

### Calculating Φ*

The Practical PHI Toolbox (https://figshare.com/articles/code/phi_toolbox_zip/3203326), a publicly available MATLAB software package, was used to implement measures derived from IIT 2.0.^13,17,24^ The measure presented in the main manuscript was Φ*, or integrated information from the decoding perspective. We obtained Φ* from the same set of binarized time-series used to obtain μ[Φ^max^]. The procedure previously applied involves computing Φ* for the minimum information partition (MIP) out of all possible symmetric bipartitions of the system.^11,18^ In our case, the networks consisted of five elements, so we evaluated all possible bipartitions into groups of 2 and 3 and recorded the minimum value obtained as Φ*.

### Calculating average correlation

For every pair of regions in each RSN, the Pearson correlation coefficient *p* was computed between the two time-series using the SciPy statistics module in Python (https://docs.scipy.org/doc/scipy/reference/stats.html). Unlike CD, one bidirectional measure was obtained for each pair. The average of all correlations, μ[*p*], was then obtained by adding up all the correlations and dividing by 10 (the total number of possible pairs).

### Permutations in the control procedures

In the control procedures, the time-series were generated by concatenating all 17 subjects for each network and condition. Then, individual nodes from each of the 11 RSNs we randomly distributed into 100 control networks, ensuring all nodes within a random network came from different RSNs. We then calculated μ[Φ^max^] and the reference metrics for each new time-series. This was repeated for every conscious condition to obtain the four control distributions shown in Fig. 2.

For the temporal control procedure, we permuted each RSN’s concatenated time-series and calculated μ[Φ^max^] for the new signals. All time points were shuffled individually, which completely reordered each time-series’ sequences of states and introduced a high level of disorder to the signals. We generated 50 shuffled time-series for each RSN and condition.

### Magnitude and reflection of awareness level

Our final analysis introduced two additional ways to analyze an RSN’s behavior in terms of a particular metric. The values of the four conditions were arranged into a four-element vector in the appropriate order:

1 $$\mathop{{{{{{\rm{M}}}}}}}\limits^{ \rightharpoonup }=[{{{{\upmu }}}}[{\Phi}^{{{\max }}}]_{{{{{{\rm{A}}}}}}}\,{{{{\upmu }}}}[{\Phi }^{{{\max }}}]_{{{{{{\rm{M}}}}}}}\,{{{\upmu }}}[{\Phi }^{{{\max }}}]_{{{{{{\rm{D}}}}}}}\,{{{\upmu }}}[{\Phi }^{{{\max }}}]_{{{{{{\rm{R}}}}}}}]$$

The overall magnitude of the network with respect to the metric was then computed by taking the square-root of the sum of all elements squared:

2 $${{{\rm{M}}}}=\sqrt{{\upmu }[{{\Phi }^{\max }}]_{{{\rm{A}}}}^{2}+{\upmu }[{{\Phi }^{\max }}]_{{{\rm{M}}}}^{2}+{\upmu }[{{\Phi }^{\max }}]_{{{\rm{D}}}}^{2}+{\upmu }[{{\Phi }^{\max }}]_{{{\rm{R}}}}^{2}}$$

We then created a simple model vector that gradually decreases moving towards deep sedation and rebounds in recovery, which represents changes to awareness level throughout anesthesia:

3 $$\mathop{{{{{{\rm{E}}}}}}}\limits^{ \rightharpoonup }=\left[1,0,-1,1\right]$$

To determine the extent to which a network’s modulation pattern reflects these changes, we computed the cosine similarity ${{{{{\rm{S}}}}}}$ between each network’s respective $\mathop{{{{{{\rm{M}}}}}}}\limits^{ \rightharpoonup }$ vector and $\mathop{{{{{{\rm{E}}}}}}}\limits^{ \rightharpoonup }$, which effectively represents the degree to which changes in $\mathop{{{{{{\rm{M}}}}}}}\limits^{ \rightharpoonup }$ correspond to changes in the model, or the degree to which the two vectors are parallel.

4 $${{{{{\rm{S}}}}}}=\frac{\mathop{{{{{{\rm{M}}}}}}}\limits^{ \rightharpoonup }\,\cdot \, \mathop{{{{{{\rm{E}}}}}}}\limits^{ \rightharpoonup }}{\left|\mathop{{{{{{\rm{M}}}}}}}\limits^{ \rightharpoonup }\right|\left|\mathop{{{{{{\rm{E}}}}}}}\limits^{ \rightharpoonup }\right|}$$

Note that the dot product represents element-by-element multiplication and addition of the two vectors. The same procedure was applied to the reference metrics (i.e., $\mathop{{{{{{\rm{M}}}}}}}\limits^{ \rightharpoonup }=[{\Phi }_{{{{{{\rm{A}}}}}}}^{* },{\Phi }_{{{{{{\rm{M}}}}}}}^{* },{\Phi }_{{{{{{\rm{D}}}}}}}^{* },\,{\Phi }_{{{{{{\rm{R}}}}}}}^{* }]$).

### Statistics and reproducibility

For the modulation results shown in Figs. 3 and 4, we created sampling distributions of μ[Φ^max^] for each RSN and condition. 17 time-series were generated by concatenating the time-series from 16 subjects and leaving a different subject out each time (i.e., first time-series: concatenate subjects 2, 3, 4, … 17; second time-series: concatenate subjects 1, 3, 4, … 17, etc.). For each of these time-series, μ[Φ^max^] and each reference metric was computed to obtain a sample of values. The mean of this sample was taken to be the mean of the population, and the standard deviation of the sample was taken to be the standard error, which was multiplied by √17 to obtain the standard deviation of the population.^64^ We tested for statistically significant differences between the four conditions using Welch’s t-test.^65^ Since t-tests between all pairs of conditions were repeated for each RSN, we used the Benjamini–Hochberg procedure to correct for multiple comparisons.^66^ More methodological details and exact statistical values are provided Supplementary Note 6.

To ensure the reproducibility of our results, we provide the clustered time-series (i.e., those including five regions), in Supplementary Data 1, as well as code used to compute μ[Φ^max^] (see Data Availability and Code Availability statements for more information).

### Statistical test for the Markov property

IIT 3.0 and PyPhi were formulated with the assumption that time series satisfy the Markov property and conditional independence. The Markov Property pertains to a time-series where the state at a particular time *t* depends only on the state of the previous time point, *t* *−* 1; dependencies on *t* *−* 2, *t* *−* 3, etc. are not allowed.^21,67^ We implemented a statistical test that analyzes the sequences of states appearing in each time-series to detect a violation of this property.^68–70^ Starting with the most commonly occurring three-state sequence (states *a, b, c; b* *≠* *c*), we recorded the occurrence of this sequence and any other three-state sequences that end with the same two states but start with a different one (i.e., a three-state sequence where the second and third states are *b*, *c*). Note that these are the states of the entire system and not individual nodes, meaning a, b, etc. are state with indices between 0 and 31. A violation of the Markov property is more likely if a particular three-state sequence occurs at a greater frequency than others that start with the same two states, or if the third state in the sequence occurs more frequently when it is preceded by the first two states.

We assigned a label *h* (*h* = *1,2, 3, …, H*) to the three-state sequences mentioned above, and the following quantities were obtained for each one: Sequence count, SC: The occurrence of the three-state sequence in the time-series; Non-sequence count, NSC: The count of all two-state sequences that match the first two states of the three-state sequence but are followed by a different third state from *h* (*a, b, e, e* *≠* *c*); Total sequence count, TSC: The count of all two-state sequences that correspond to the first two states of the three-state sequence, including those followed by the third state in the sequence of interest (*a, b, c; a, b, e; TSC* = *SC* + *NSC)*. These quantities were then organized into a contingency table, the layout of which is given in Table 2. Each recorded count is referred to as *N*_*hk*_*. The* index *h* corresponds to rows, and the index *k* corresponds to columns (1 or 2 for *SC or NSC*), *N*_*hT*_ represents a row total, *N*_*Tk*_ represents a column total, and *n* represents the total of all entries in the table.

**Table 2:** Contingency table layout for the Markov property test.

*h*	*SC*	*NSC*	*TSC*
1	*N*_*11*_	*N*_*12*_	*N*_*1T*_
2	*N*_*21*_	*N*_*21*_	*N*_*2T*_
3	*N*_*31*_	*N*_*32*_	*N*_*3T*_
.	.	.	.
.	.	.	.
.	.	.	.
*H*	*N*_*H1*_	*N*_*H2*_	*N*_*HT*_
Total	*N*_*T1*_	*N*_*T2*_	*n*

The following contingency table was constructed to compare the most common three-state sequence to other sequences that share the same states. Rows correspond to a specific three-state sequence. The columns correspond to counts of the three-state sequences and the occurrences of the first two states within them. The values in the table were used to compute a χ^2^ statistic, which indicated the extent to which the time-series deviates from the Markov property.

The table was used to compute a χ^2^ value, which indicates the extent to which the time-series deviates from the Markov property.^71^ This can be seen as a comparison between the sequence distribution of the time-series to that of a sequence distribution that satisfies the Markov property (i.e., the goodness of fit of a Markovian distribution to that of the time-series). The following equation was used to compute χ^2^ from the contingency table:

5 $${\chi }^{2}=\mathop {\sum }\limits_{h,k}\frac{{\left[{N}_{{hk}}-n\left({N}_{{hT}}/n\right)\left({N}_{{Tk}}/n\right)\right]}^{2}}{n\left({N}_{{hT}}/n\right)\left({N}_{{Tk}}/n\right)}$$

The value obtained was then used to obtain a *p* value with SciPy. Significance was set to 0.05 and the number of degrees of freedom was *H* *−* 1. Any significant result indicated a significant deviation from the sequence distribution of a Markov chain, and hence a violation of the Markov property. We repeated this test for the time-series obtained by concatenating all subjects. The results obtained for each RSN’s time-series (with 17 subjects concatenated) are presented in Supplementary Note 5; Supplementary Table 2.

### Test for conditional independence

For every state-by-node TPM, there is one unique state-by-state TPM that is conditionally independent, which is outputted by PyPhi when converting a TPM from state-by-node to state-by-state form. We checked the extent to which our data met this property by taking the original state-by-state TPM, converting it to state-by-node, and then back to state-by-state. The relative distance *D* between the original TPM (*A*) and the conditionally independent TPM (*B*) was computed by subtracting the two arrays and calculating the Frobenius norm of the residual, which was divided by the Frobenius norm of the conditionally independent variant.

6 $$D=\frac{\sqrt{\mathop{\sum}\limits_{i}\mathop{\sum}\limits_{j}{\left|{A}_{i,j}-{B}_{i,j}\right|}^{2}}}{\sqrt{\mathop{\sum}\limits_{i}\mathop{\sum}\limits_{j}{\left|{B}_{i,j}\right|}^{2}}}$$

Results for the conditional independence test are provided in Supplementary Note 5; Supplementary Table 1.

### Reporting summary

Further information on research design is available in the Nature Portfolio Reporting Summary linked to this article.

## Supplementary information

Supplementary Information

Description of Additional Supplementary Files

Supplementary Data 1

Supplementary Data 2

Supplementary Data 3

Supplementary Data 4

Reporting Summary

## Data Availability

The raw imaging dataset used in this study is available in Openneuro.org (https://openneuro.org/datasets/ds003171). All processed time-series, which were used as inputs for our calculations, are provided in Supplementary Data 1. Supplementary Data 2 contains source data for Fig. 2, Supplementary Data 3 contains the source data for Figs. 3 and 4, and Supplementary Data 4 was used to generate Fig. 5.

## Code availability

The PyPhi module was the principal tool used to compute integrated information (https://pyphi.readthedocs.io/en/latest/). The primary functions used to compute integrated information, which were developed using the PyPhi module, have been published with open access on Zenodo.org (https://zenodo.org/record/8033892).^72^ The core functions are also shown and explained in Supplementary Note 2.
