# Supplementary material for: An implementation of integrated information theory in resting-state fMRI
Source: Commun Biol. 2023 Jul 5;6:692. doi: 10.1038/s42003-023-05063-y (PMC10322831; doi:10.1038/s42003-023-05063-y)
Supplement: Supplementary file 1 — Supplementary Information [file 42003_2023_5063_MOESM1_ESM.pdf]

Supplementary Material for

"An Implementation of Integrated Information Theory in Resting- State fMRI"

Idan E. Nemirovsky, Nicholas J.M. Popiel, Jorge Rudas, Matthew Caius,
Lorina Naci, Nicholas D. Schiff, Adrian M. Owen, Andrea Soddu

Table of Contents

Section	Title	
1	Supplementary Note 1: Cortical Regions in Resting-State Networks	1
2	Supplementary Note 2: Code Listing for Computing $\mu[\Phi^{max}]$	2
3	Supplementary Note 3: Modulation by Sedative and the Reference Metrics	4
4	Supplementary Note 4: Mechanism Level Integrated Information	7
5	Supplementary Note 5: Markov Property and Conditional Independence Tests	9
7	Supplementary Note 6: Statistical Results for Comparisons	11
8	Supplementary Note 7: Grouped Permutations	17
9	References	23

Supplementary Note 1: Cortical Regions in Resting-State Networks

Following the parcellation method described in the main manuscript[1], a k-means algorithm was applied to reduce each RSN's number of regions to five. This number was chosen to maintain good balance between computational complexity of Φ^{max} and each network's and spatial resolution. The five regions obtained for all 11 RSNs with this method are presented in Supplementary Figure 1.

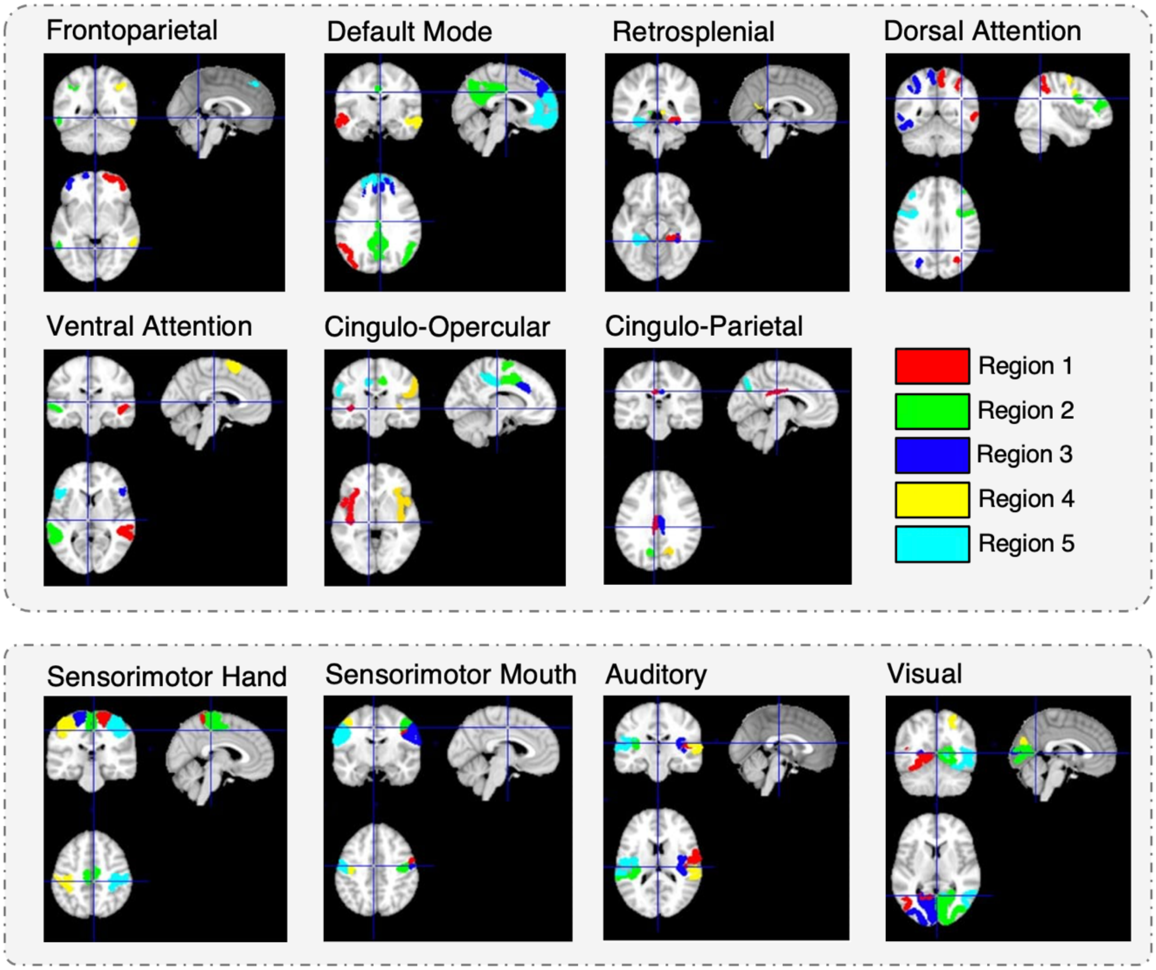

Supplementary Figure 1. Representative cortical regions of the 11 resting-state networks (RSNs) from the parcellation scheme. After the k-means procedure, each RSN consisted of five regions, which was chosen to balance spatial resolution with computational efficiency. The first seven networks are those associated with higher-order function, whereas the bottom panel contains sensorimotor and sensory networks.

Supplementary Note 2: Code Listing for Computing $\mu[\Phi^{max}]$

To compute $\mu[\Phi^{max}]$, the first step was to generate a state-by-state TPM directly from the time-series. This was accomplished with the `build_tpm` function, which counts through all transitions occurring in the inputted time-series and populates the TPM accordingly. Note that PyPhi[2] requires indexing and binary-to-integer conversion to follow little-endianness (LE). Transition counts are normalized with respect to the total number of transitions out of a particular state, which are used obtain transition probabilities. The second output (`weights_ts`) gives the percent frequency of each state, which was obtained by counting the total number of occurrences in the time-series and dividing by the total number of time points.

```
def build_tpm(time_series):

    # Inputs:
    # 1) time_series  (Array with dimensions n_time_points X n_regions)
    #
    # Outputs:
    # 1) tpm (Array with dimensions n_states X n_states)
    #       Each entry gives the probability of transition between two states.
    #
    # 2) weights (Array with dimensions 1 X n_states)
    #       Each entry gives the probability of a state appearing in the time-series.

    import numpy as np
    import pyphi

    # Obtain bi time-series.
    avgs = np.mean(time_series, axis=0)

    time_series_copy = np.copy(time_series)

    for i in range(len(avgs)):
        time_series[np.where(time_series_copy[:, i] >= avgs[i]), i] = 1
        time_series[np.where(time_series_copy[:, i] < avgs[i]), i] = 0

    time_series = time_series.astype(np.int)

    markov_chain = time_series.tolist()
    n = len(markov_chain[0])
    tpm = np.zeros((2 ** n, 2 ** n))

    # Loop through all transitions and populate TPM.
    for (s1, s2) in zip(markov_chain, markov_chain[1:]):
        i = pyphi.convert.state2le_index(s1)
        j = pyphi.convert.state2le_index(s2)
        tpm[i][j] += 1

    # Create array for transition counts.
    transitions_total = np.sum(tpm, axis= -1)

    # Normalize counts in TPM to obtain probabilities.
    for div in range(len(transitions_total)):
        if transitions_total[div] != 0.0:
            tpm[div, :] /= transitions_total[div]

    # Create array for state counts.
    weights_ts = np.zeros((2 ** time_series.shape[-1]))

    for s in markov_chain:
        i = pyphi.convert.state2le_index(s)
        weights_ts[i] += 1

    weights_ts /= len(markov_chain)

    return np.copy(tpm), np.copy(weights_ts)
```

The next function used was `mean_phi_max`, which takes the TPM and weights array as inputs. For each state appearing in the time-series, Φ^{max} is obtained and multiplied by the corresponding weight of the state. Note that since the TPMs might not meet the conditional independence property, we converted the TPM outputted by `build_tpm` to state-by-node form (`tpm_sbn`) using `pyphi.convert`:

```
tpm_sbn = pyphi.convert.to_2dimensional(pyphi.convert.state_by_state2state_by_node(tpm))
```

A complete analysis typically involves evaluating Φ for every possible subset of elements and finding Φ^{max} from the one that maximizes integrated information. The function below was used to obtain a list of all possible subsets for a given number of elements (`n`). The output (`subset_list`) ranges from all subsets that include two elements to the subset including all elements.

```
def all_subsets(n):

    from itertools import combinations
    subset_list = []

    for n_i in range(n + 1):
        sub = list(combinations(range(n), n_i))

        if len(sub[0]) > 1:
            for s in sub:
                subset_list.append(s)

    return subset_list
```

Initially, we conducted a complete subset analysis over the time-series for networks with all subjects concatenated. We found that Φ was always maximized for the subset that includes all of a networks' regions, and in subsequent analyses (i.e., the control procedures and grouped permutations), we evaluated Φ over this subset only. The `subset_type` input can be changed from `"All"` to `"Full"`, which restricts the calculation to the subset that includes the entire network.

```
def mean_phi_max(tpm_sbn, weights_ts, subset_type = "All"):

    # Inputs:
    # 1) tpm_sbn (State-by-node form, dimensions n_states X n_regions)
    # 2) weights (Array with dimensions 1 X n_states)
    #
    # 3) subset_type (Type of subset analysis)
    #       "All" : Find phi_max for from all possible subsets
    #       "Full": Find phi for the subset including all network elements
    #
    # Outputs:
    # 1) phi_weight
    #       Weighted average of phi_max for the TPM's corresponding time-series.

    import numpy as np
    import pyphi
    from pyphi.compute import phi

    rows, columns = tpm_sbn.shape
    setting_int = np.linspace(0, rows - 1, num=rows).astype(int)
    M = list(map(lambda x: list(pyphi.convert.le_index2state(x, columns)), setting_int))
    M = np.asarray(M).astype(np.int)

    phi_values = []
    network = pyphi.Network(tpm_sbn)

    for state in range(rows):
        if weights_ts[state] != 0:

            if subset_type == "Full":
                phi_values.append(phi(pyphi.Subsystem(network, M[state, :], range(network.size))))

            elif subset_type == "All":
                phi_subset_list = []
                subset_list = all_subsets(columns)

                for s in subset_list:

                    phi_subset_list.append(phi(pyphi.Subsystem(network, M[state, :], s)))

                phi_values.append(max(phi_subset_list))

    weights_non_zero = weights_ts[np.where(weights_ts != 0)]
    mean_phi = np.sum(phi_values * weights_non_zero)

    return mean_phi
```

Supplementary Note 3: Modulation by Sedative and the Reference Metrics

Integrated Information from the Decoding Perspective

To calculate integrated information from the decoding perspective (Φ*)[3], we employed the Practical PHI Toolbox (https://figshare.com/articles/code/phi_toolbox_zip/3203326). As with the equivalent version of these results for μ[Φ^{max}] in the main manuscript, we generated 17 time-series by concatenating 16 subjects and leaving a different subject out each time. The procedure used involved finding the minimum information partition (MIP), or the partition that minimizes Φ*, over all possible symmetric bipartitions of the system.[4,5] This means that Φ* was computed over all possible cuts of the system into two parts consisting of 2 and 3 elements, which was as symmetric as possible for a system consisting of five elements.

Finding the MIP ensures that if the system consists of independent subsystems, Φ* is evaluated over the least interdependent parts (i.e., the MIP provides the most "natural" way to partition the system). The results are shown in Supplementary Figure 2, where we give absolute values of Φ* and statistical significances with respect to the awake condition.

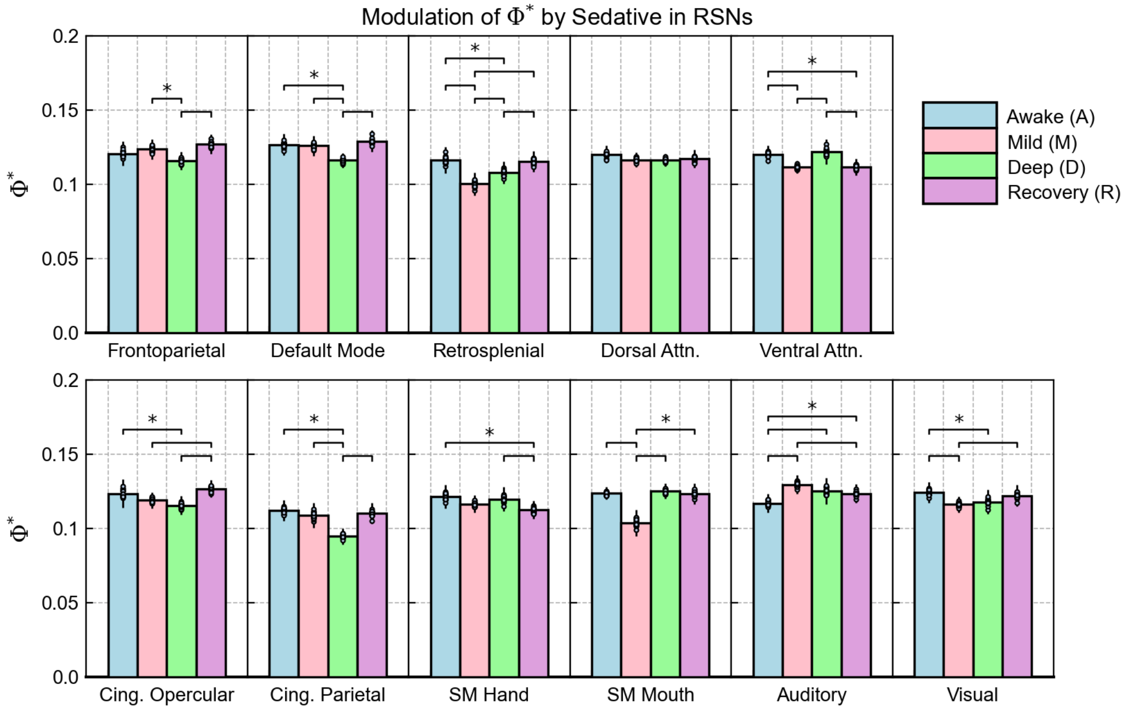

Supplementary Figure 2. Modulation of Φ* individual RSNs over symmetric bipartitions. For each network, the average integrated information is compared for the four conditions of awareness. Bar heights represent the mean of the distribution obtained by concatenating 16 subjects with a different subject left out each time (N = 17, mean ± SD, with error bars representing SD = √17 * SE, SE being the standard error of the sample mean). All statistically significant differences found between the conditions (i.e., awake vs. deep sedation) are indicated by the horizontal lines above the bars: *; $p < \alpha^{BH}$, where α^{BH} is the significance threshold obtained with the Benjamini-Hochberg correction for multiple comparisons between conditions: A vs. M; α^{BH} = 0.023, A vs. D; α^{BH} = 0.027, A vs. R; α^{BH} = 0.014, M vs. D; α^{BH} = 0.027, M vs. R; α^{BH} = 0.023; D vs. R; α^{BH} = 0.032.

Unlike μ[Φ^{max}], only the cingulate networks reflected the conscious evolution of subjects (i.e., awake > mild > deep < recovery). Moreover, the overall variability of this measure across different networks was found to be low compared to the other measures.

Causal Density

For causal density (CD), we used the Multivariate Granger Causality Toolbox (https://www.mathworks.com/matlabcentral/fileexchange/78727-the-multivariate-granger-causality-mvgc-toolbox).[6] The Granger causality was computed for every pair of regions and CD was obtained by averaging over all values. The results for each network are shown in Supplementary Figure 3. Overall, the RSNs present a variety of behaviours through this measure, with some similarities to the results seen for μ[Φ^{max}]. Four networks closely reflected the conscious evolution of subjects, which were the frontoparietal, default mode, dorsal attention, and retrosplenial networks. On the other hand, the sensory and sensorimotor networks show no consistent modulation pattern.

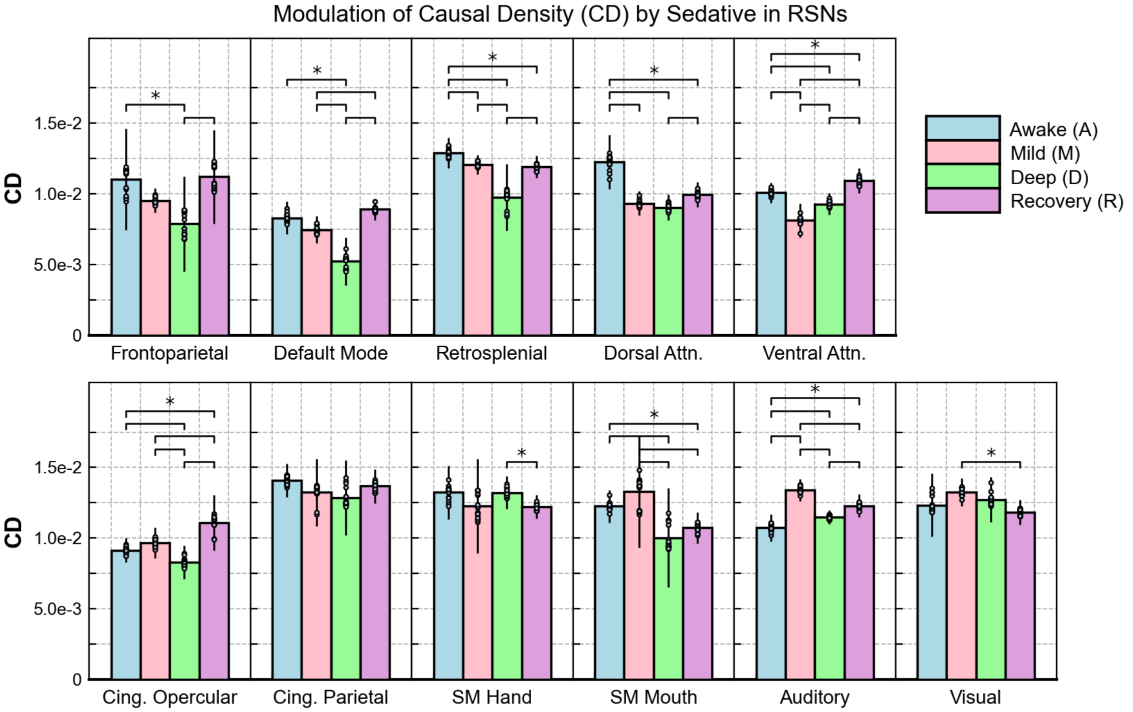

Supplementary Figure 3. Modulation of CD individual RSNs over symmetric bipartitions. For each network, causal density (CD) is compared for the four conditions of awareness. Bar heights represent the mean of the distribution obtained by concatenating 16 subjects with a different subject left out each time (N = 17, mean ± SD, with error bars representing SD = √17 * SE, SE being the standard error of the sample mean). All statistically significant differences found between the conditions (i.e., awake vs. deep sedation) are indicated by the horizontal lines above the bars: *; $p < \alpha^{BH}$, where α^{BH} is the significance threshold obtained with the Benjamini-Hochberg correction for multiple comparisons between conditions: A vs. M; α^{BH} = 0.018, A vs. D; α^{BH} = 0.036, A vs. R; α^{BH} = 0.027, M vs. D; α^{BH} = 0.027, M vs. R; α^{BH} = 0.027; D vs. R; α^{BH} = 0.036.

Average Pearson Correlation Coefficient

In contrast to the modulation of $\mu[\Phi^{max}]$, which varied substantially over the collection of RSNs, $\mu[\rho]$ presents a homogeneous behaviour that reflects the conscious state of subjects in most networks. In the discussion of the main manuscript, we argue that while it may appear as though correlations are a more reliable measure of conscious level, it is well-established that certain cortical networks are more important for conscious processing than others. Accordingly, we should expect a measure of conscious level to behave differently over different cortical regions, with some regions being more strongly affected than others. In other words, the globalized behaviour of $\mu[\rho]$ does not allow for differentiation of cortical regions based on modulation pattern.

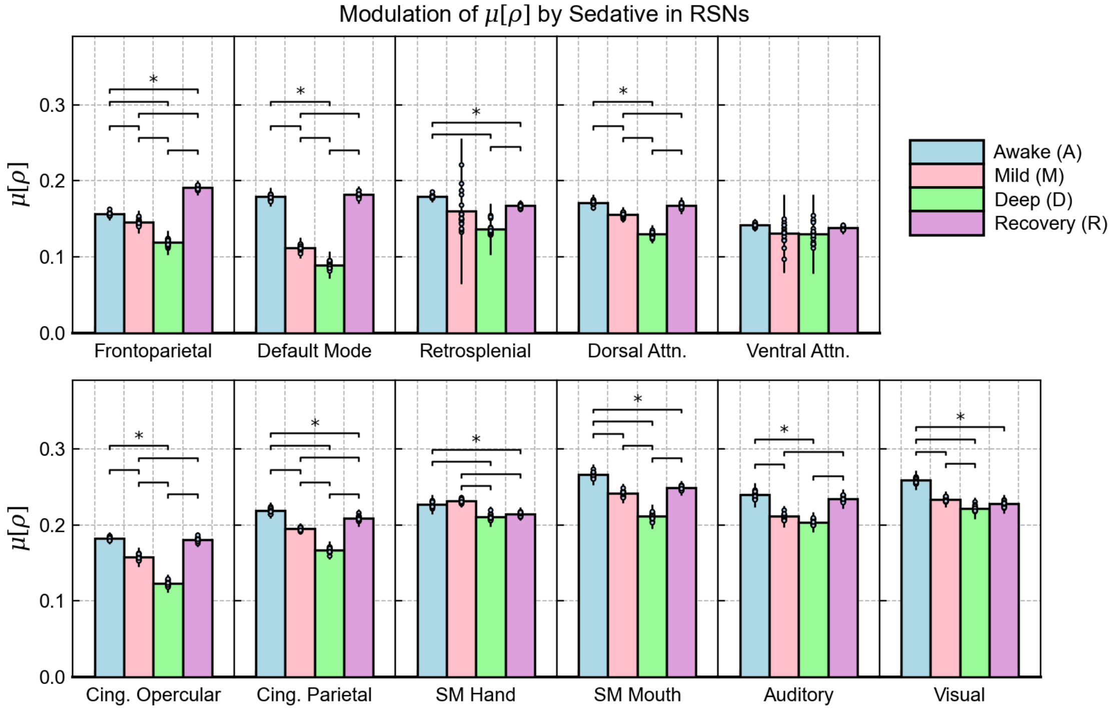

Supplementary Figure 4. Modulation of $\mu[\rho]$ by propofol in individual RSNs. For each network, the average Pearson correlation coefficient is compared for the four conditions of awareness. Bar heights represent the mean of the distribution obtained by concatenating 16 subjects with a different subject left out each time (N = 17, mean ± SD, with error bars representing SD = √17 * SE, SE being the standard error of the sample mean). All statistically significant differences found between the conditions (i.e., awake vs. deep sedation) are indicated by the horizontal lines above the bars: *; $p < \alpha^{BH}$, where α^{BH} is the significance threshold obtained with the Benjamini-Hochberg correction for multiple comparisons between conditions: A vs. M; α^{BH} = 0.036, A vs. D; α^{BH} = 0.045, A vs. R; α^{BH} = 0.027, M vs. D; α^{BH} = 0.036, M vs. R; α^{BH} = 0.032; D vs. R; α^{BH} = 0.036.

Supplementary Note 4: Mechanism Level Integrated Information

A potential solution to deal with computation cost of Φ^{max} is to evaluate the integrated information of individual mechanisms (ϕ).[7,8] In this analysis, we computed the mechanism-level integrated information and compared its results with those of $\mu[\Phi^{max}]$. To obtain variation related specifically to mechanisms, we used the time-series obtained by concatenating all 17 subjects. We chose to focus only on high-order mechanisms (i.e., mechanisms consisting of 2 or more elements) because there is a large discrepancy between ϕ for higher-order and elementary mechanisms (i.e., those consisting of individual elements). A TPM was constructed using the same approach described in the main manuscript, and we computed mechanism-level integrated information as follows:

1. A set of 2 or more elements was used to define the mechanism (i.e., nodes 0 & 1).
2. For each network's time-series, ϕ was computed for the mechanism using PyPhi; like $\mu[\Phi^{max}]$, this involved obtaining a weighted average of the mechanisms ϕ value for every state appearing in the time-series, which was used to obtain $\mu[\phi]$ for a particular mechanism.
3. $\mu[\phi]$ was computed for every other possible mechanism (i.e., nodes 0 & 2, nodes 0 & 3, … nodes 0, 1, 2, … nodes 0, 1, 2, 3, … nodes 0, 1, 2, 3, 4).
4. $\langle\mu[\phi]\rangle$ was obtained by averaging over the $\mu[\phi]$ values of all higher-order mechanisms.

The results for every RSN and condition are shown in Supplementary Figure 5 below.

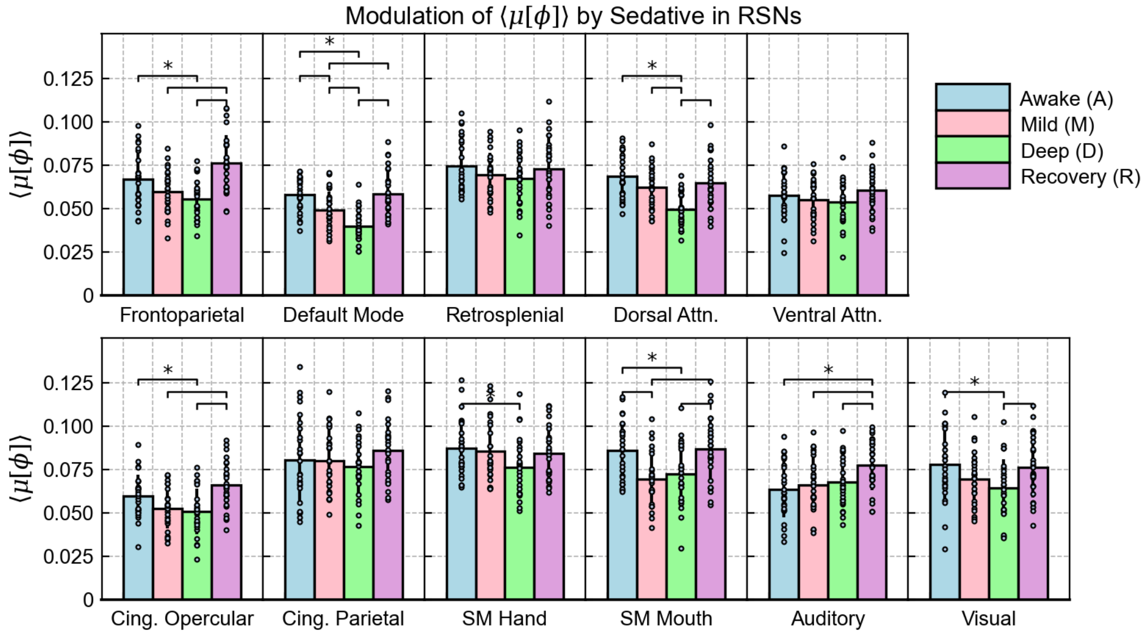

Supplementary Figure 5. Modulation of $\langle\mu[\phi]\rangle$ by propofol in individual RSNs. For each network's concatenated time-series, we computed a weighted average of integrated information for each mechanism, ($\mu[\phi]$) and then averaged over all possible mechanisms (N = 28 mechanisms, mean ± SD, with error bars representing SD). All statistically significant differences found between the conditions (i.e., awake vs. deep sedation) are indicated by the horizontal lines above the bars: *; $p < \alpha^{BH}$, where α^{BH} is the significance threshold obtained with the Benjamini-Hochberg correction for

multiple comparisons between conditions: A vs. M; α^{BH} = 0.014, A vs. D; α^{BH} = 0.032, A vs. R; α^{BH} = 0.005, M vs. D; α^{BH} = 0.01, M vs. R; α^{BH} = 0.023; D vs. R; $\alpha^{BH} = 0.032$.

First, we note that some networks behave differently through this measure of integrated information as compared to $\mu[\Phi^{max}]$. The frontoparietal (FPN) and dorsal attention (DAN) networks still present a modulation pattern that reflects the conscious evolution of subjects with significant changes. However, this is also the case for more networks, including the default mode, cingulate-opercular, sensorimotor, and visual networks. This means that for certain networks, an analysis over individual mechanisms may not be substantial to estimate $\mu[\Phi^{max}]$.

As mentioned, the mechanism-based metric does not go beyond computing the integrated information of individual mechanisms. To understand the difference in these results, it is important to consider how Φ^{max} with respect to this counterpart: 1) the integrated information ("small phi", or ϕ) of each mechanism is computed; 2) each mechanism, its ϕ value, and cause-effect repertoires are used to specify a concept; 3) each concept is used to populate a high dimensional space called a conceptual structure; 4) Φ^{max} is computed by partitioning the conceptual structure and measuring its irreducibility.[9,10] In other words, Φ^{max} measures the integration of a conceptual structure, which according to IIT, is a complete representation of a conscious experience. On the other hand, ϕ measures the integration of concepts, which are parts of the conscious experience but do not represent consciousness in its entirely.

Since concepts are derived directly from specific mechanisms, Φ^{max} does not only depend on the integration of each mechanism, but also on how strongly the mechanisms are integrated with respect to one another, making it a much more thorough measure of integration than ϕ. Even if a network's mechanisms yield high ϕ, a set of independent mechanisms will result in low Φ^{max}. On the other hand, if its mechanisms are collectively irreducible through the conceptual structure, Φ^{max} will be high.

Our results can therefore be explained by the idea that Φ^{max} goes several steps beyond ϕ to describe a network's integration. While several networks closely reflect changes to awareness level through $\langle\mu[\phi]\rangle$, only two (the FPN and DAN) demonstrate this property through $\mu[\Phi^{max}]$. Considering the differences discussed between these two measures, the final conclusion we make is that with the data we obtained and the procedure we applied, only the FPN and DAN reflect changes to conscious level via the full set of criteria specified by IIT 3.0.

Supplementary Note 5: Markov Property and Conditional Independence Tests

To determine the extent to which a TPM was conditionally independent, we computed the relative residual between a raw TPM (generated directly from the time-series) and its conditionally independent counterpart (obtained through PyPhi processing) as described in the Methods of the main manuscript. The Frobenius norm of the residual matrix was computed, which was divided by the Frobenius norm of the conditionally independent matrix.[9] The results are presented in Supplementary Table 1, where the deviation between the two matrices is given as a percent difference.

In summary, this test showed that the relative difference between the two matrices ranges from 20% to 80% depending on the network and condition in question. The RSNs whose transition matrices most closely resemble their conditionally independent counterparts are the Default Mode Network (Awake: 38%, Mild: 28%, Deep: 22%, Recovery: 41%) Frontoparietal Network (45%, 35%, 35%, 54%), and Dorsal Attention Network (43%, 39%, 35%, 45%).

These results indicate that the fMRI signals we obtained do not completely satisfy the conditional independence property. Calculations of Φ^{max} are based on the conditionally independent variant of the TPM, and as a consequence of converting to this form, some of the features contained in the original signals are lost. Nevertheless, we believe that the mechanisms preserved in the conditionally independent variants can still be used to meaningfully calculate integrated information, especially for networks with lower percent differences.

	**Awake**	**Mild**	**Deep**	**Recovery**
Frontoparietal	36.97	27.48	21.81	39.22
Default Mode	43.49	33.75	34.46	51.52
Retrosplenial	57.36	50.75	49.34	59.69
Dorsal Attention	41.56	37.85	34.53	43.9
Ventral Attention	42.94	33.44	35.98	40.5
Cingulo-Opercular	42.27	33.93	31.14	41.9
Cingulo-Parietal	67.85	68	56.85	65.12
SM Hand	51.53	62.68	60.34	56.74
SM Mouth	68.59	61.28	51.44	64.93
Auditory	62.24	53.76	54.73	65.36
Visual	74.88	62.24	60	71.37

Supplementary Table 1. Conditional independence test. Percent differences between the TPMs generated directly from the time-series and their conditionally independent counterparts are presented in this table. RSNs are given across rows, and the four conditions are presented in columns.

Turning to the Markov property, nearly all time-series acquired by concatenating the entire subject population did not show statistically significant differences that indicate a violation of Markovian behaviour. Out of the 44 time-series tested (11 RSNs, 4 conscious states), three yielded $p < 0.05$: the retrosplenial network in the recovery condition ($p = 0.03$), the ventral attention network in the awake condition ($p = 0.04$), and the cingulo-parietal network in the awake condition ($p = 0.02$). For all relevant statistics, see Supplementary Table 2.

	χ^2	ν	χ^2/ν	p	
Frontoparietal	35.66	28	1.27	0.15	*Awake*
	22.25	28	0.79	0.77	*Mild*
	29.24	29	1.01	0.45	*Deep*
	10.52	30	0.35	1.00	*Recovery*
Default Mode	23.16	28	0.83	0.72	
	16.46	27	0.61	0.94	
	29.59	28	1.06	0.38	
	25.46	30	0.85	0.70	
Retrosplenial	21.94	29	0.76	0.82	
	10.85	22	0.49	0.98	
	29.33	29	1.01	0.45	
	41.82	27	1.55	**0.03**	
Dorsal Attn.	16.13	28	0.58	0.96	
	17.68	30	0.59	0.96	
	15.43	25	0.62	0.93	
	8.52	28	0.30	1.00	
Ventral Attn.	34.41	22	1.56	**0.04**	
	34.48	23	1.50	0.06	
	22.99	24	0.96	0.52	
	16.57	29	0.57	0.97	
Cing. Operc.	8.18	31	0.26	1.00	
	13.33	30	0.44	1.00	
	21.72	31	0.70	0.89	
	11.35	29	0.39	1.00	
Cing. Parietal	45.11	27	1.67	**0.02**	
	27.28	29	0.94	0.56	
	19.34	29	0.67	0.91	
	15.09	30	0.50	0.99	
SM Hand	22.43	30	0.75	0.84	
	33.61	31	1.08	0.34	
	42.94	31	1.39	0.08	
	31.18	29	1.08	0.36	
SM Mouth	26.26	30	0.88	0.66	
	32.52	29	1.12	0.30	
	38.78	30	1.29	0.13	
	22.70	29	0.78	0.79	
Auditory	22.76	31	0.73	0.86	
	41.19	29	1.42	0.07	
	17.43	30	0.58	0.97	
	20.10	31	0.65	0.93	
Visual	17.34	31	0.56	0.98	
	24.75	30	0.82	0.74	
	36.24	31	1.17	0.24	
	21.78	30	0.73	0.86	

Supplementary Table 2. Results for the Markov property contingency table test. Each block corresponds to an RSN, with four rows corresponding to the four states of awareness (awake, mild, deep, recovery). The first column gives the χ^2 values obtained from equation 5 of the main manuscript, the second column gives the degrees of freedom ν (number of sequences – 1), the third column gives a reduced χ^2 value, or χ^2/ν, and the fourth column gives p values. Time-series yielding statistically significant differences ($p < 0.05$) were considered to violate the Markov property and their p-values are highlighted with red text.

Supplementary Note 6: Statistical Results for Condition Comparisons

Statistical Test for the Control Procedure

In the control procedures, we tested whether the original (unpermuted) networks fell within the control distributions. This is essentially a test to verify that permutations did indeed impose a significant decrease on $\mu[\Phi^{max}]$ or the reference metrics.

For each condition, the spatial control sample was generated by randomly grouping regions from different networks 100 times (N = 100), whereas the temporal control distribution was generated by permuting each RSN's signal randomly 50 times (For 11 RSNs and 50 permutations, N = 550). The two control distributions were then standardized, and each original RSN's $\mu[\Phi^{max}]$ value (or that of the reference metrics) was assigned a z-score with respect to both control distributions:

$$z_{\text{net}} = \frac{\mu[\Phi^{\text{max}}]_{\text{net}} - \mu_{\text{control}}}{\sigma_{\text{control}}} \qquad (1)$$

Using each network's z-score, a p value was then calculated with significance set to 0.05. Supplementary Table 3, we show which networks that fell significantly within the spatial and temporal control distributions ($p \geq 0.05$) for each of the metric analyzed.

Statistical Test for Analysis of Modulation by Sedative

For our evaluation of sedative-induced changes to each network's $\mu[\Phi^{max}]$, we concatenated 16 of the 17 subjects and left a different subject out for each time-series. The mean of this sampling distribution was taken to be the mean of the population μ, and the standard deviation of the sample was defined as the standard error of the sample mean. The standard error was multiplied by $\sqrt{(N = 17)}$ to obtain the standard deviation of the population (σ). For the same network, two of the four conditions i and j were compared using Welch's t-test[11], which is used for comparisons of two samples with different variances. For every possible pair of conditions, the t values were computed as follows:

$$t = \frac{\mu_i - \mu_j}{\sqrt{\frac{1}{N}({\sigma_i}^2 - {\sigma_j}^2)}} \qquad (2)$$

We then computed the degrees of freedom using the Welch-Satterthwaite equation[11] (simplified for two samples of the same size):

$$\nu = (N-1)\frac{({\sigma_i}^2 + {\sigma_j}^2)^2}{{\sigma_i}^4 + {\sigma_j}^4} \qquad (3)$$

Finally, we obtained a two-tailed p - value using t and ν. With four conditions, there were a total of six possible comparisons for each network. All t, ν, and p values obtained for each RSN's modulation are presented in Supplementary Tables 4 and 5.

Results for the Control Procedures

As shown in Figure 2 of the main manuscript, the metric with the least amount of sensitivity to *both* spatial and temporal permutations was Φ*. This is evident in the table, as it had the most networks that fell within the control distributions. With the exception of the DMN in some conditions, $\mu[\Phi^{max}]$ and CD distinguished the time-series of nearly all original RSNs from their permuted counterparts. While correlations dropped significantly in the spatial control procedure, they did not change as a result of temporal permutations.

**$\mu[\Phi^{max}]$**	**Awake**	**Mild**	**Deep**	**Recovery**
Spatial Control	DMN; **(1)**	(0)	DMN; **(1)**	(0)
Temporal Control	(0)	(0)	(0)	(0)
**CD**	**Awake**	**Mild**	**Deep**	**Recovery**
Spatial Control	(0)	(0)	DMN; **(1)**	(0)
Temporal Control	(0)	(0)	(0)	(0)
**Φ***	**Awake**	**Mild**	**Deep**	**Recovery**
Spatial Control	Retro, CP, Auditory; **(3)**	DAN, VAN, CO, CP, SM Hand, Visual; **(6)**	FPN, DMN, CO, DAN, Retro, Visual; **(6)**	Retro, VAN, CP, SM Hand; **(4)**
Temporal Control	FPN, DMN, Retro, DAN, VAN, CO, CP, SM Hand, SM Mouth, Visual, Auditory; **(11)**	FPN, DMN, DAN, VAN, CO, CP, SM Hand, SM Mouth, Visual; **(9)**	FPN, DMN, Retro, DAN, VAN, CO, CP, SM Hand, SM Mouth, Visual, Auditory; **(10)**	Retro, DAN, VAN, CP, SM Hand, Visual, Auditory; **(7)**
**$\mu[\rho]$**	**Awake**	**Mild**	**Deep**	**Recovery**
Spatial Control	(0)	DMN; **(1)**	DMN, FPN; **(2)**	(0)
Temporal Control	N.A.			

Supplementary Table 3. Networks falling within spatial and temporal control distributions. For integrated information and each reference metric, we list the networks for which values were not significantly different from those obtained in the control procedures. With respect to the control distributions, the networks listed had a *p* value equal to or greater than 0.05. The number of networks without significance is reported in each box. For each condition, N = 100 for the spatial control distributions and N = 550 (repeated 50 times for each RSN) for the temporal control distributions. Temporal permutations had no effect on correlations, hence the N.A. label in the final row.

Results for Modulation of $\mu[\Phi^{max}]$

Frontoparietal

	M	D	R	
A	1.93	4.74	–4.45	t
	29.44	26.53	31.35	ν
	0.063	<1E-05	0.0001	p
M		2.74	–5.19	
		30.96	31.23	
		0.01	<1E-05	
D			-7.94	
			28.83	
			<1E-05	

Dorsal Attention

	M	D	R
A	1.92	8.6	4.02
	30.27	31.85	29.22
	0.065	0	0.00037
M		5.47	1.99
		29.31	31.82
		<1E-05	0.055
D			–2.96
			28.15
			0.0062

Retrosplenial

	M	D	R
A	–0.18	0.61	–0.22
	31.46	31.67	31.99
	0.86	0.54	0.83
M		0.85	–0.05
		31.97	31.32
		0.4	0.96
D			–0.84
			31.56
			0.41

Cingulo-Opercular

	M	D	R
A	0.93	0.49	0.45
	28.28	20.88	30.55
	0.36	0.63	0.66
M		–0.77	–0.53
		25.62	31.23
		0.45	0.6
D			0.05
			23.55
			0.96

Ventral Attention

	M	D	R
A	–0.09	1.29	1.41
	31.87	31.42	30.53
	0.93	0.21	0.17
M		1.34	1.45
		30.8	26.69
		0.19	0.16
D			–0.31
			30.46
			0.76

Cingulo-Parietal

	M	D	R
A	1.25	1.38	0.67
	30.89	31.15	31.61
	0.22	0.18	0.24
M		0.16	1.58
		31.98	31.89
		0.87	0.12
D			–1.67
			28.39
			0.1

Default Mode

	M	D	R
A	–0.39	2.29	–1.7
	30.53	31.03	31.51
	0.7	0.029	0.099
M		3.01	–1.5
		31.94	28.78
		0.0051	0.15
D			–3.95
			29.44
			0.00045

Supplementary Table 4. Results for statistical comparison of $\mu[\Phi^{max}]$ over the four states of awareness in higher-order networks. (A: awake; M: mild; D: Deep; Recovery: R). Welch's t-test was used to obtain t, ν (degrees of freedom), and p values, which are given in this respective order within each comparison block (highlighted with the bolded borders). Positive t values indicate a decrease of $\mu[\Phi^{max}]$, whereases negative values indicate an increase.

Sensorimotor Mouth

		M	*D*	*R*
A	t	1.14	0.28	−0.6
	ν	31.77	30.99	30.41
	p	0.26	0.78	0.55
M	t		−0.95	−1.61
	ν		31.7	29.22
	p		0.35	0.12
D	t			−0.88
	ν			27.71
	p			0.39

Sensorimotor Hand

		M	*D*	*R*
A	t	−0.34	2.58	1.19
	ν	31.91	30.04	31.6
	p	0.74	0.015	0.24
M	t		2.92	1.58
	ν		29.27	31.89
	p		0.0067	0.12
D	t			−1.67
	ν			28.39
	p			0.1

Visual

		M	*D*	*R*
A	t	0.27	−1.27	– 0.04
	ν	24.02	32	27.79
	p	0.79	0.21	0.97
M	t		– 1.1	– 0.27
	ν		24.11	30.24
	p		0.28	0.79
D	t			0.95
	ν			27.89
	p			0.35

Auditory

		M	*D*	*R*
A	t	2.4	– 1.95	– 2.42
	ν	28.4	28.42	31.86
	p	0.023	0.061	0.021
M	t		– 3.73	– 4.25
	ν		32	28.76
	p		0.00073	0.00017
D	t			– 0.06
	ν			29.43
	p			0.95

Supplementary Table 5. Results for statistical comparison of $\mu[\Phi^{max}]$ over the four states of awareness in sensorimotor and sensory RSNs. (A: awake; M: mild; D: Deep; Recovery: R). Welch's t-test was used to obtain t, ν (degrees of freedom), and p values, which are given in this respective order within each comparison block (highlighted with the bolded borders). Positive t values indicate a decrease of $\mu[\Phi^{max}]$, whereases negative values indicate an increase.

Correction for Multiple Comparisons in Results for $\mu[\Phi^{max}]$

Since the comparison for each pair of conditions (i.e., awake vs. mild sedation, etc.) was repeated for each of the 11 networks, we applied a correction for multiple comparisons to the *p*-values obtained in Supplementary Tables 6 and 7. The Benjamini-Hochberg procedure[12,13] was applied as follows:

1) The *p*-values for each condition comparison were ranked from smallest to largest and assigned a rank number (k) from 1 to 11.
2) The null hypothesis (H_o) was defined to be the case where $\mu[\Phi^{max}]$ does not differ significantly between a pair of conditions.
3) The original α level ($\alpha = 0.05$) was adjusted via multiplication by the rank and division by the number of networks (m).
4) The *p*-values were compared to the adjusted α values; the significance threshold was set at the highest k value (k_{max}) for which a network's p-value was less than α (k / m). H_o was rejected for all networks with $k \leq k_{max}$.

Awake vs. Mild

Network	p	k	α (k / m)	Reject H_o
Auditory	0.023	1	0.0045	False
Frontoparietal	0.063	2	0.0091	False
Dorsal Attn.	0.065	3	0.014	False
Cing. Parietal	0.22	4	0.018	False
SM Mouth	0.26	5	0.023	False
Cing. Operc.	0.36	6	0.027	False
Default Mode	0.70	7	0.032	False
SM Hand	0.74	8	0.036	False
Visual	0.79	9	0.041	False
Retrosplenial	0.86	10	0.045	False
Ventral Attn.	0.93	11	0.050	False

Awake vs. Recovery

Network	p_i	k	α (k / m)	Reject H_o
Frontoparietal	0.00010	1	0.0045	True
Dorsal Attn.	0.00037	2	0.0091	True
Auditory	0.021	3	0.014	False
Default Mode	0.099	4	0.018	False
Ventral Attn.	0.17	5	0.023	False
SM Hand	0.24	6	0.027	False
Cing. Parietal	0.51	7	0.032	False
SM Mouth	0.55	8	0.036	False
Cing. Operc.	0.66	9	0.041	False
Retrosplenial	0.83	10	0.045	False
Visual	0.97	11	0.050	False

Awake vs. Deep

Network	p_i	k	α (k / m)	Reject H_o
Dorsal Attn.	<1E-05	1	0.0045	True
Frontoparietal	<1E-05	2	0.0091	True
SM Hand	0.015	3	0.014	False
Default Mode	0.029	4	0.018	False
Auditory	0.061	5	0.023	False
Cing. Parietal	0.18	6	0.027	False
Ventral Attn.	0.21	7	0.032	False
Visual	0.21	8	0.036	False
Retrosplenial	0.54	9	0.041	False
Cing. Operc.	0.63	10	0.045	False
SM Mouth	0.78	11	0.050	False

Supplementary Table 6. Multiple comparison correction for $\mu[\Phi^{max}]$ for comparisons involving the awake condition. For each possible comparison between the four conditions of awareness, statistical significance was determined by applying a multiple comparison correction to the *p*-values associated with Welch's t-test (Supplementary Tables 4 and 5). This was done using the Benjamini-Hochberg procedure, where networks were arranged in terms of increasing *p*-value, assigned a rank k, and then compared to the adjusted threshold α (k / m), with m being the number of times a comparison was repeated (i.e., the number of networks).

Mild vs. Deep

Network	p_i	k	α (k / m)	Reject H_0
Dorsal Attn.	<1E-05	1	0.0045	True
Auditory	0.00073	2	0.0091	True
Default Mode	0.0051	3	0.014	True
SM Hand	0.0067	4	0.018	True
Frontoparietal	0.010	5	0.023	True
Ventral Attn.	0.19	6	0.027	False
Visual	0.28	7	0.032	False
SM Mouth	0.35	8	0.036	False
Retrosplenial	0.40	9	0.041	False
Cing. Operc.	0.45	10	0.045	False
Cing. Parietal	0.87	11	0.050	False

Mild vs. Recovery

Network	p_i	k	α (k / m)	Reject H_0
Frontoparietal	<1E-05	1	0.0045	True
Auditory	0.00017	2	0.0091	True
Dorsal Attn.	0.055	3	0.014	False
SM Mouth	0.12	4	0.018	False
SM Hand	0.12	5	0.023	False
Default Mode	0.15	6	0.027	False
Ventral Attn.	0.16	7	0.032	False
Cing. Operc.	0.60	8	0.036	False
Cing. Parietal	0.62	9	0.041	False
Visual	0.79	10	0.045	False
Retrosplenial	0.96	11	0.050	False

Deep vs. Recovery

Network	p_i	k	α (k / m)	Reject H_0
Frontoparietal	0.023	1	0.0045	True
Default Mode	0.00045	2	0.0091	True
Dorsal Attn.	0.0062	3	0.014	True
SM Hand	0.10	4	0.018	False
Visual	0.35	5	0.023	False
SM Mouth	0.39	6	0.027	False
Retrosplenial	0.41	7	0.032	False
Cing. Parietal	0.53	8	0.036	False
Ventral Attn.	0.94	9	0.041	False
Auditory	0.95	10	0.045	False
Cing. Operc.	0.96	11	0.050	False

Supplementary Table 7. Multiple comparison correction for $\mu[\Phi^{max}]$ for comparisons involving other conditions. For each possible comparison between the four conditions of awareness, statistical significance was determined by applying a multiple comparison correction to the *p*-values associated with Welch's t-test (Supplementary Tables 4 and 5). This was done using the Benjamini-Hochberg procedure, where networks were arranged in terms of increasing *p*-value, assigned a rank *k*, and then compared to the adjusted threshold α (k / m), with *m* being the number of times a comparison was repeated (i.e., the number of networks).

Supplementary Note 7: Grouped Permutations

Analysis for $\mu[\Phi^{max}]$

The following analysis is an extension of our investigation into the BOLD signal's temporal properties and how they relate to integrated information.

As seen in the temporal control procedures, the BOLD signal's inherent causality can be captured with integrated information. This was underscored by the observation that $\mu[\Phi^{max}]$ is subject to a ten-fold decrease when the order of its state-transitions is disrupted. While the control procedure consisted of shuffling time points individually, the following analysis extended this idea by permuting each network's time-series in grouped blocks of time points.

The order of time points within these blocks was unchanged, but the blocks themselves were rearranged in random order. This was repeated for blocks of several sizes *n*, including 2, 3, 5, 7, 15, and 35 time points, which allowed for varying degrees of fluctuation based on the number of time points in each block. As an intermediate step between the single time point permutations and groups of 2, we also permuted in groups of 2 and 1, where two time points were grouped, and the next point was permuted individually ($n = 1.5$). For each *n*, we permuted to obtain 20 new time-series, from which we obtained the means and standard deviations of $\mu[\Phi^{max}]$ as shown in Supplementary Figures 6 and 7. This allowed us to analyze how $\mu[\Phi^{max}]$ is affected with varying degrees of disruption to the original time-series. Different networks are presented across rows, while the four conditions are presented in the columns.

The general behaviour demonstrated in all cases is the same: as *n* increases, $\mu[\Phi^{max}]$ converges towards the values of the original time-series, from which the $n = 35$ distributions do not deviate significantly in most cases. This result is not surprising considering the nature of the permutations performed; with smaller *n*, there is a larger number of blocks to be shuffled, which induces a greater degree of sequential disruption and yields time-series with more random transitions. On the other hand, permutations with larger *n* swap fewer transitions and hence preserve more of the original signal.

Interestingly, it appears that the length of these plateaus, and hence the rate at which the ordered $\mu[\Phi^{max}]$ value is approached, varies across networks and conditions. To describe these changes quantitatively, the plots in Supplementary Figures 6 and 7 were fitted using an exponential plateau function:

$$\mu[\Phi^{\max}] = \Gamma[1 - e^{-\lambda n}] \qquad (4)$$

The first parameter, Γ, corresponds to the maximum value reached by the curve and is effectively an approximation of the network's unpermuted $\mu[\Phi^{max}]$, while the exponential parameter λ is proportional to the rate at which the plateau is approached. More specifically, a high λ corresponds to a faster rate of approach as the number of grouped time points increases, which can be observed with a longer plateau (i.e., $\mu[\Phi^{max}]$ is near the maximum at $n = 5$). In contrast, networks with low λ demonstrate a steadier increase that continues with larger *n* values, and their plateau is visibly shorter. The values obtained for this parameter are presented in the fifth columns of Supplementary Figures 6 and 7.

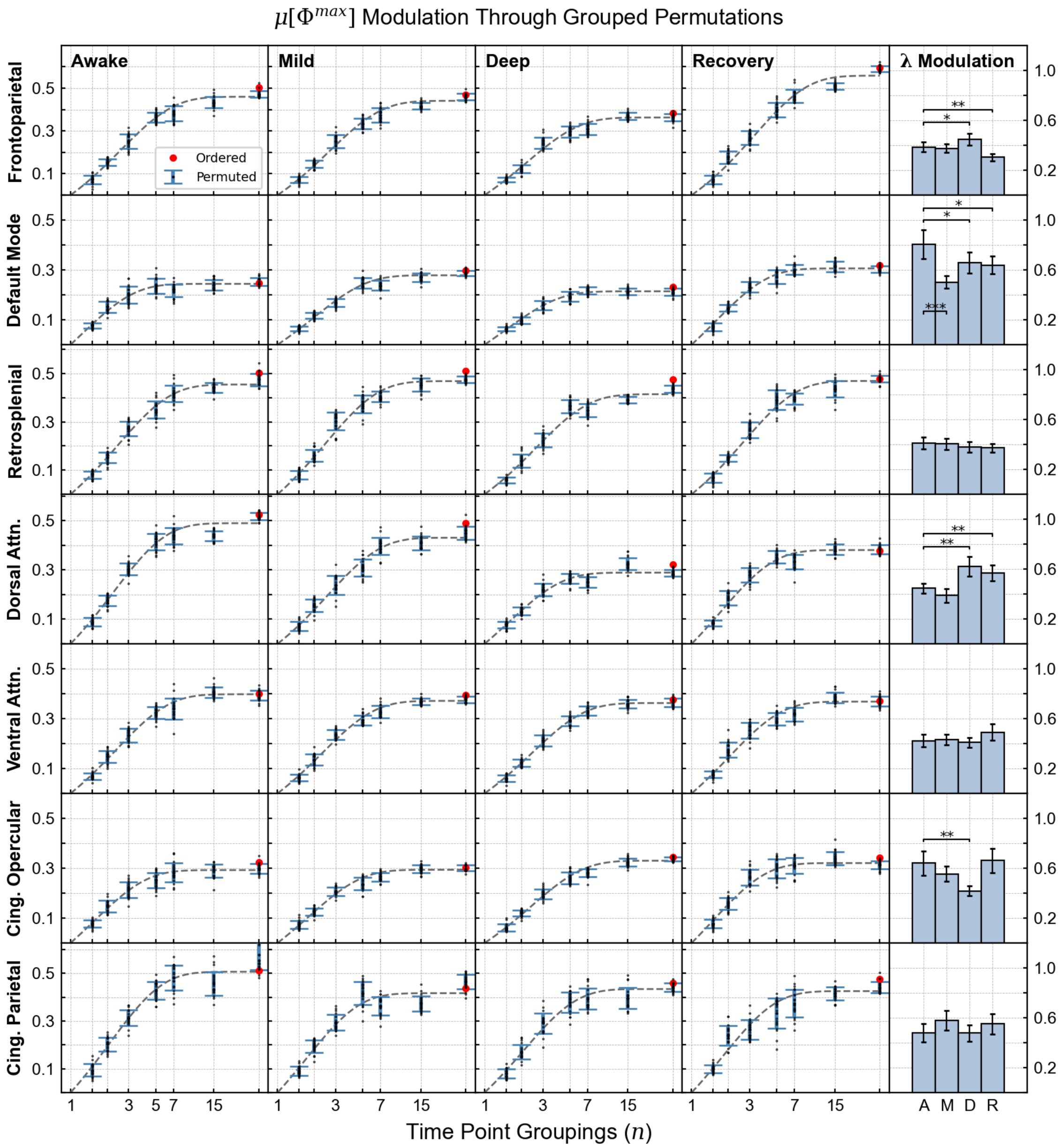

Supplementary Figure 6. Grouped permutations in higher-order RSNs. For several group sizes containing n time points, permutations were performed by randomly rearranging the positions of grouped blocks within the time-series. $\mu[\Phi^{max}]$ is plotted against each n (logarithmically scaled) for higher-order RSNs. RSNs are given along rows and the four conditions are presented across columns. The graphs were fitted using an exponential plateau function, from which we obtained the exponential parameter λ to quantify the rate of approach towards the plateau with respect to increasing n. For each RSN, the modulation of λ over the four conditions is shown in the fifth column. Standard deviations for λ were obtained based on the variance of the $\mu[\Phi^{max}]$ values used for fitting. Statistically significant differences are shown only for comparisons that involve the awake condition ($0.01 < p < 0.05$: ∗, $0.001 < p < 0.01$: ∗∗, $p < 0.001$: ∗∗∗). Error bars in the first four columns correspond to the sample standard deviation of the permuted samples (N = 20 for each n), while error bars in the fifth column represent the standard deviation of the fit for each condition. Note that a single value was obtained for each condition's fit parameter, and its standard deviation was derived from that of the data points used to fit it.

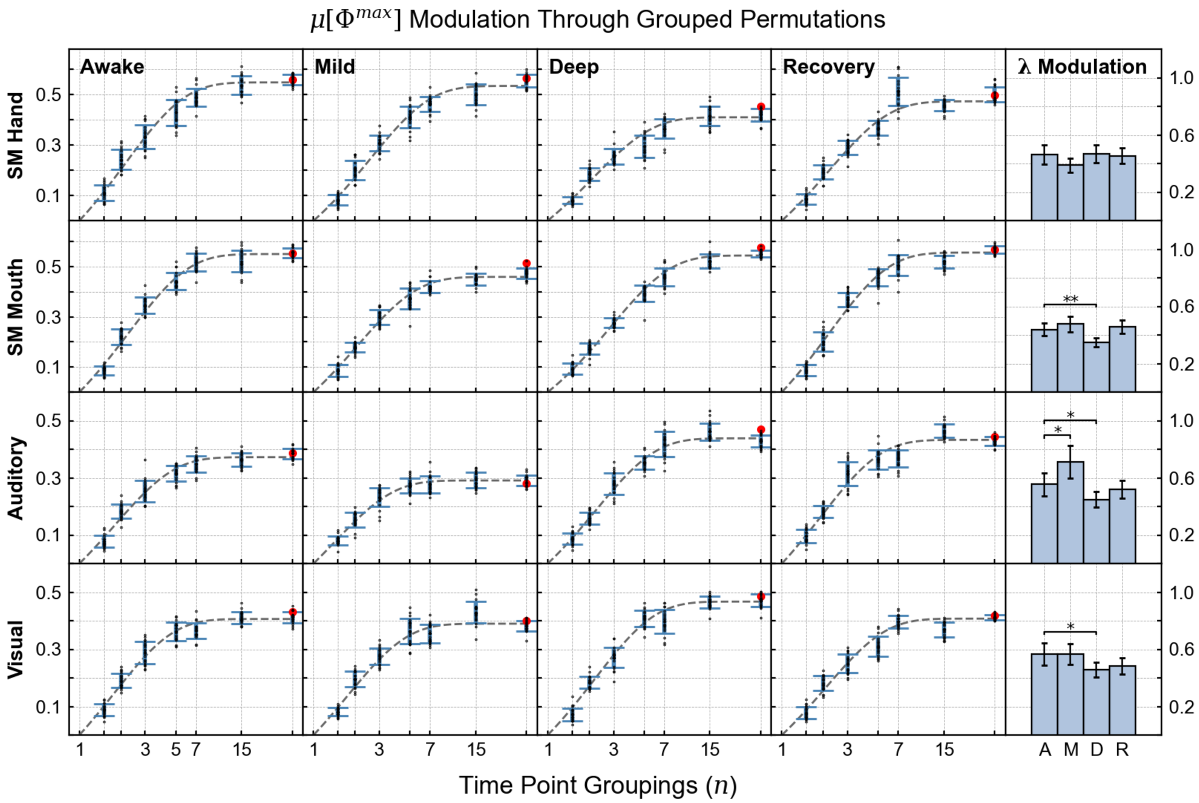

Supplementary Figure 7. Grouped permutations in sensorimotor and sensory RSNs. The results for the same procedure are shown but for RSNs associated with sensory and motor functions. Error bars in the first four columns correspond to the sample standard deviation of the permuted samples (N = 20 for every *n*), and error bars in the fifth column represent the standard deviation of the fit for each condition. Note that a single value was obtained for each condition's fit parameter, and its standard deviation was derived from that of the data points used to fit it.

Upon closer inspection, it is noticeable that higher λ values pertain to networks lower in $\mu[\Phi^{max}]$. For example, consider the DMN and FPN in the awake condition (Supplementary Figure 6, rows 1 & 2): the latter generates $\mu[\Phi^{max}]$ nearly twice greater than the former at the plateau, while its λ values are substantially lower (DMN: $\mu[\Phi^{max}] = 0.30$, $\lambda = 0.81$; frontoparietal: $\mu[\Phi^{max}] = 0.56$, $\lambda = 0.39$). The same observation holds true for other RSNs higher in $\mu[\Phi^{max}]$, such as the retrosplenial, ventral-attention, and mouth sensorimotor networks. This relationship is also apparent for sedative-induced changes within the same network. In the FPN and DAN, $\mu[\Phi^{max}]$ decreased moving towards deep sedation and increased in recovery; in terms of λ, both networks demonstrate a significant increase in deep sedation followed by a significant decrease in recovery. In the FPN, $\mu[\Phi^{max}]$ peaked during the recovery phase, which corresponds to a significant dip and minimum value for λ in the fourth condition. Taking all networks and conditions together, the Pearson correlation coefficient between $\mu[\Phi^{max}]$ and λ is –0.68 (p = 1e–5, with 44 samples for each parameter), indicating a moderate to strong negative correlation.

Some higher-order RSNs, most notably the ventral and retrosplenial networks, presented no significant changes in $\mu[\Phi^{max}]$ throughout sedation. This is also the case for their λ values, which do not fluctuate significantly over the four conditions. Furthermore, there appears to be an exception to this trend in the DMN; although λ is significantly different for the four conditions, it presents no clear relationship

with $\mu[\Phi^{max}]$. The hand sensorimotor cortex presents another exception, as there are no significant changes in λ despite substantial variations in its $\mu[\Phi^{max}]$. Otherwise, the remaining sensory and motor networks also demonstrate an inverse relationship between the two parameters.

Since larger *n* values correspond to more preservation of the original time-series, λ effectively represents the capacity of a signal to maintain its integrated information despite disruptions to its temporal structure. In networks with high λ and low $\mu[\Phi^{max}]$, grouped permutations do not drastically impact $\mu[\Phi^{max}]$ because the signals contain fewer causal structures that generate integrated information. This is also apparent from their rapid approach towards the plateau, which shows that the dynamics giving rise to $\mu[\Phi^{max}]$ can be captured over a block containing as few as five time points. On the other hand, highly integrated networks with low λ are more susceptible to permutations; their time-series contain more "consequential" transitions that contribute to $\mu[\Phi^{max}]$. Permutations with larger *n* can significantly reduce their integrated information, which indicates a more sophisticated temporal behaviour spanning a larger group of time points.

In other words, λ represented a time-series' capacity to withstand permutations, and this was stronger in networks where integrated information was low. On the other hand, the low λ values of highly integrated networks reflected a more elaborate temporal structure; their time-series contained a higher proportion of "consequential" transitions that contributed to $\mu[\Phi^{max}]$, which accounts for their higher susceptibility to permutations. These differences were also observed for different conditions within the same network, which provided insight into the anaesthetic's effects on resting-state temporal dynamics.

Analysis for **CD**

Since the control procedure showed that Causal Density (CD) is also strongly reduced when time-points are permuted, we wanted to test whether the time-series will behave in a similar way for this metric when permuted in groups. Thus, we computed CD for the same set of time-series with grouped permutations and applied the same type of fit (see Supplementary Figures 8 and 9).

While CD also presents a similar behaviour with increasing group size, its λ values were substantially smaller than those of $\mu[\Phi^{max}]$, meaning that the plateau is approached more steadily for CD (i.e., the graphs present a "shorter" plateau). Moreover, the correlation between this fit parameter and CD was – 0.47 (p =1e-3), which was lower than its correlation with $\mu[\Phi^{max}]$.

One major difference is that while this metric also presents a plateau-like behaviour, the CD values of the original RSNs are still significantly higher than those corresponding to the least disruptive permutation (n = 35). This means that the values obtained for CD depend more on each time-series in its entirety, which is in line with the vector autoregressive (VAR) model used to compute Granger causality. On the other hand, $\mu[\Phi^{max}]$ is computed from a transition probability matrix (TPM), which was constructed using the count of transitions between states in a time-series, and this does not strongly depend on the order of the entire signal. For this reason, these results show that the dependence of CD on an entire signal made it more sensitive to grouped permutations than $\mu[\Phi^{max}]$.

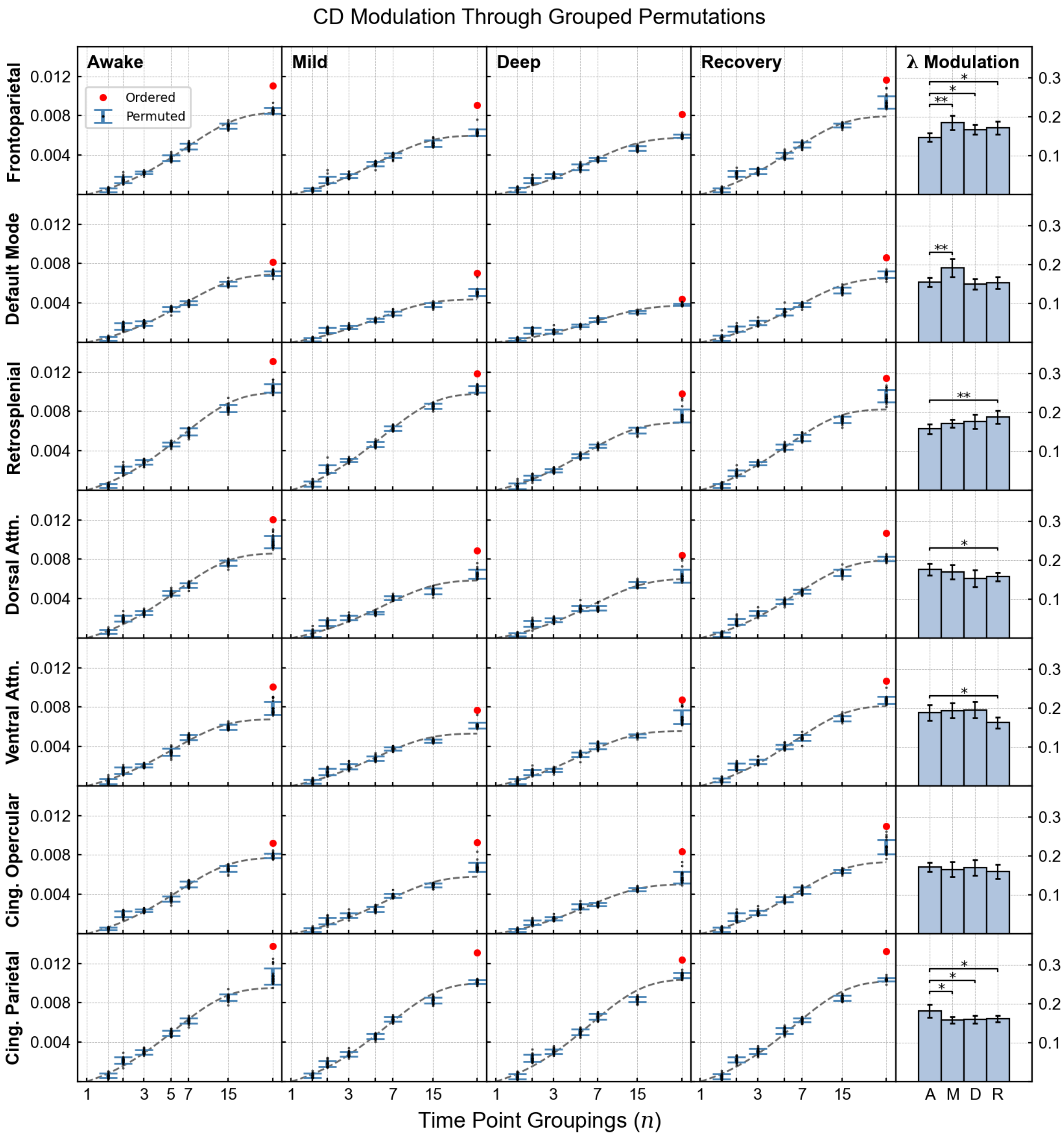

Supplementary Figure 8. Grouped permutations and their effects on CD in higher-order RSNs. The mean and standard deviation of CD (20 values) are plotted against each n (logarithmically scaled) for the RSNs that are associated with higher-order functions. The graphs were fitted using an exponential plateau function, from which we obtained the parameter λ to quantify the rate of approach towards the plateau with respect to increasing n. Statistically significant differences are shown for comparisons that involve the awake condition ($0.01 < p < 0.05$: $*$, $0.001 < p < 0.01$: $**$, $p < 0.001$: $***$). Error bars in the first four columns correspond to the sample standard deviation of the permuted samples (N = 20 for every n), and error bars in the fifth column represent the standard deviation of the fit for each condition. Note that a single value was obtained for each condition's fit parameter, and its standard deviation was derived from that of the data points used to fit it.

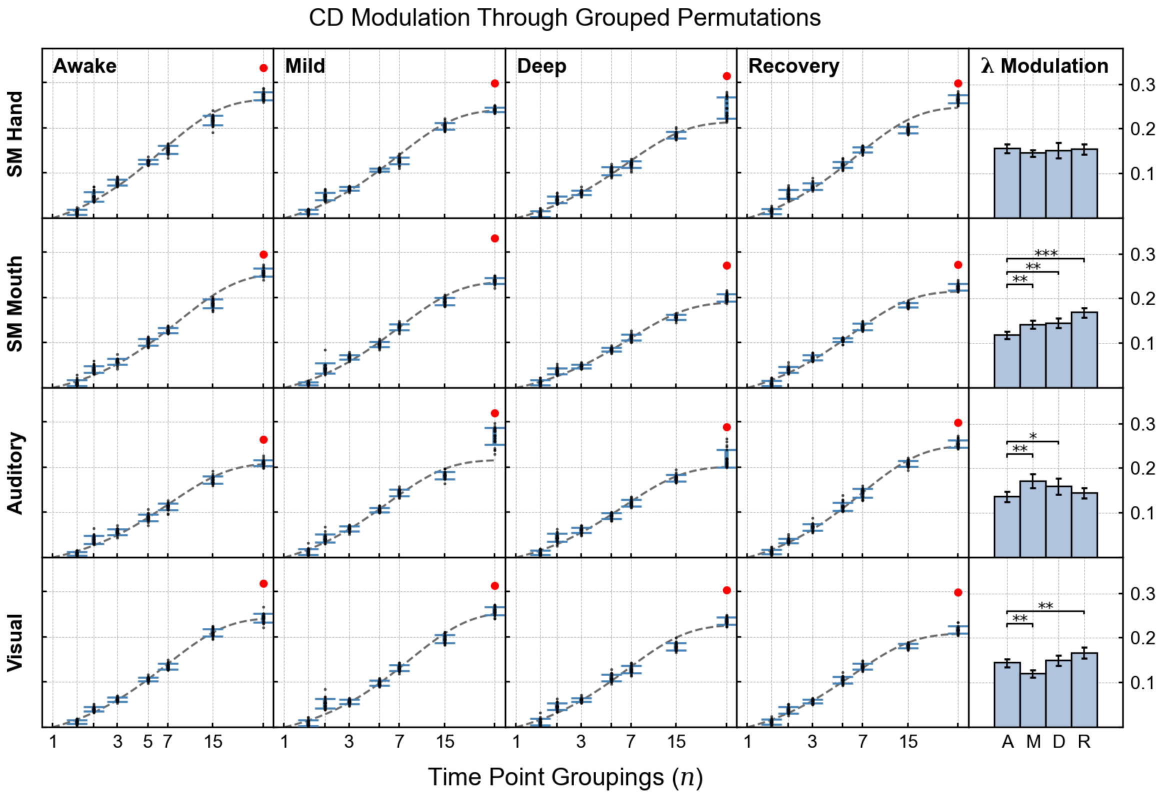

Supplementary Figure 9. Grouped permutations and their effects on CD in sensorimotor and sensory RSNs. The results for the same procedure are shown but for RSNs associated with sensory and motor functions. Error bars in the first four columns correspond to the sample standard deviation of the permuted samples (N = 20 for every n), and error bars in the fifth column represent the standard deviation of the fit for each condition. Note that a single value was obtained for each condition's fit parameter, and its standard deviation was derived from that of the data points used to fit it.

Supplementary References

1. Gordon EM, Laumann TO, Adeyemo B, Huckins JF, Kelley WM, Petersen SE. Generation and Evaluation of a Cortical Area Parcellation from Resting-State Correlations. *Cerebral Cortex*. 2016;26(1):288-303. doi:10.1093/cercor/bhu239
2. Mayner WGP, Marshall W, Albantakis L, Findlay G, Marchman R, Tononi G. PyPhi: A toolbox for integrated information theory. *PLoS Comput Biol*. 2018;14(7):e1006343. doi:10.1371/journal.pcbi.1006343
3. Oizumi M, Amari S ichi, Yanagawa T, Fujii N, Tsuchiya N. Measuring Integrated Information from the Decoding Perspective. *PLoS Comput Biol*. 2016;12(1):e1004654. doi:10.1371/journal.pcbi.1004654
4. Boly M, Sasai S, Gosseries O, et al. Stimulus Set Meaningfulness and Neurophysiological Differentiation: A Functional Magnetic Resonance Imaging Study. *PLoS One*. 2015;10(5):e0125337. doi:10.1371/journal.pone.0125337
5. Balduzzi D, Tononi G. Integrated Information in Discrete Dynamical Systems: Motivation and Theoretical Framework. *PLoS Comput Biol*. 2008;4(6):e1000091. doi:10.1371/journal.pcbi.1000091
6. Seth AK. A MATLAB toolbox for Granger causal connectivity analysis. *J Neurosci Methods*. 2010;186(2):262-273. doi:10.1016/j.jneumeth.2009.11.020
7. Barbosa LS, Marshall W, Albantakis L, Tononi G. Mechanism Integrated Information. *Entropy*. 2021;23(3):362. doi:10.3390/e23030362
8. Gomez JD, Mayner WGP, Beheler-Amass M, Tononi G, Albantakis L. Computing Integrated Information (Φ) in Discrete Dynamical Systems with Multi-Valued Elements. *Entropy*. 2020;23(1):6. doi:10.3390/e23010006
9. Oizumi M, Albantakis L, Tononi G. From the Phenomenology to the Mechanisms of Consciousness: Integrated Information Theory 3.0. *PLoS Comput Biol*. 2014;10(5):e1003588. doi:10.1371/journal.pcbi.1003588
10. Tononi G, Boly M, Massimini M, Koch C. Integrated information theory: from consciousness to its physical substrate. *Nat Rev Neurosci*. 2016;17(7):450-461. doi:10.1038/nrn.2016.44
11. Ruxton GD. The unequal variance t-test is an underused alternative to Student's t-test and the Mann–Whitney U test. *Behavioral Ecology*. 2006;17(4):688-690. doi:10.1093/beheco/ark016
12. Benjamini Y, Hochberg Y. Controlling the False Discovery Rate: A Practical and Powerful Approach to Multiple Testing. *Journal of the Royal Statistical Society: Series B (Methodological)*. 1995;57(1):289-300. doi:10.1111/j.2517-6161.1995.tb02031.x
13. Thissen D, Steinberg L, Kuang D. Quick and Easy Implementation of the Benjamini-Hochberg Procedure for Controlling the False Positive Rate in Multiple Comparisons. *Journal of Educational and Behavioral Statistics*. 2002;27(1):77-83. doi:10.3102/10769986027001077
