# Supplementary material for: An implementation of integrated information theory in resting-state fMRI
Source: Commun Biol. 2023 Jul 5;6:692. doi: 10.1038/s42003-023-05063-y (PMC10322831; doi:10.1038/s42003-023-05063-y)
Supplement: Supplementary file 2 — Description of Additional Supplementary Files [file 42003_2023_5063_MOESM2_ESM.pdf]

Description of Additional Supplementary Files for "An Implementation of Integrated Information Theory in Resting-State fMRI"

File Name and Type	Description
Supplementary Data 1 (zip folder)	The time-series used to compute $\mu[\Phi^{max}]$ and the reference metrics, after all processing steps and k-means clustering into five regions, are provided in this folder. Each time-series is provided as a .csv file. There are four main folders titled "Awake", "Mild", "Deep", and "Recovery" for each of the subjects' conscious states. Each of these folders includes subfolders for each of the 11 networks, which contain 17 time-series corresponding to each subject.
Supplementary Data 2 (xlsx table)	The values used to generate **Figure 2** (main manuscript) are provided in this file. The first four tabs (sub-sheets) correspond to the original, unpermuted values of $\mu[\Phi^{max}]$ (IIT3), Φ^* (IIT2), CD, and $\mu[\rho]$ (Functional Connectivity, FC). These values were obtained by concatenating all 17 subjects into single time-series for each network***** and condition. In the figure, these are given as the grey scatters. The next set of tabs correspond to the values obtained from the temporal and spatial permutations, which in the figure are given as the purple and blue violin distributions, respectively. Two tabs are included for each metric (one for each of the temporal and spatially permuted values). The exception is FC, which only includes one tab for the spatial control because temporal permutations did not change correlations.
Supplementary Data 3 (xlsx table)	The values used to generate **Figures 3 and 4** (main manuscript) and **Supplementary Figures 2, 3, and 4** are provided in this file. The metrics provided are $\mu[\Phi^{max}]$ (IIT3), Φ^* (IIT2), CD, and $\mu[\rho]$ (FC). For each metric, there are four tabs corresponding to subjects' conscious condition (Awake, Mild, Deep, Recovery). The networks are organized along columns. There are 17 values for each network; these values were obtained from concatenating the time-series of all but one of the subjects and leaving a different subject out for each measurement.
Supplementary Data 4 (xlsx table)	The values used to generate **Figure 5** (main manuscript) are provided in this file. These were generated using the data in Supplementary Data 3, and hence contain the same number of data points. Two sheets are provided for each metric, one for each network's overall magnitude (described as M in the main manuscript) and the other corresponding to the degree of modulation (described as D in the main manuscript).

*Network names are abbreviated as: **FPN**; frontoparietal, **DMN**; Default mode, **RS**; Retrosplenial, **DAN**; Dorsal attention, **VAN**; Ventral attention, **CO**; Cing. Opercular, **CP**; Cing. Parietal, **SM-H**; Sensorimotor hand; **SM-M**; Sensorimotor mouth, **VIS**; Visual, **AUD**; Auditory.
