# Supplementary material for: An implementation of integrated information theory in resting-state fMRI
Source: Commun Biol. 2023 Jul 5;6:692. doi: 10.1038/s42003-023-05063-y (PMC10322831; doi:10.1038/s42003-023-05063-y)
Supplement: Supplementary file 7 — Reporting Summary [file 42003_2023_5063_MOESM7_ESM.pdf]

nature portfolio

Corresponding author(s): Idan E. Nemirovsky

Last updated by author(s): 13/06/2023

Reporting Summary

Nature Portfolio wishes to improve the reproducibility of the work that we publish. This form provides structure for consistency and transparency in reporting. For further information on Nature Portfolio policies, see our Editorial Policies and the Editorial Policy Checklist.

Statistics

For all statistical analyses, confirm that the following items are present in the figure legend, table legend, main text, or Methods section.

n/a	Confirmed	
☐	☒	The exact sample size (*n*) for each experimental group/condition, given as a discrete number and unit of measurement
☐	☒	A statement on whether measurements were taken from distinct samples or whether the same sample was measured repeatedly
☐	☒	The statistical test(s) used AND whether they are one- or two-sided<br>*Only common tests should be described solely by name; describe more complex techniques in the Methods section.*
☒	☐	A description of all covariates tested
☐	☒	A description of any assumptions or corrections, such as tests of normality and adjustment for multiple comparisons
☐	☒	A full description of the statistical parameters including central tendency (e.g. means) or other basic estimates (e.g. regression coefficient) AND variation (e.g. standard deviation) or associated estimates of uncertainty (e.g. confidence intervals)
☐	☒	For null hypothesis testing, the test statistic (e.g. *F*, *t*, *r*) with confidence intervals, effect sizes, degrees of freedom and *P* value noted<br>*Give P values as exact values whenever suitable.*
☒	☐	For Bayesian analysis, information on the choice of priors and Markov chain Monte Carlo settings
☒	☐	For hierarchical and complex designs, identification of the appropriate level for tests and full reporting of outcomes
☒	☐	Estimates of effect sizes (e.g. Cohen's *d*, Pearson's *r*), indicating how they were calculated

Our web collection on statistics for biologists contains articles on many of the points above.

Software and code

Policy information about availability of computer code

Data collection	The PyPhi module was the principal tool used to compute integrated information (https://pyphi.readthedocs.io/en/latest/).
Data analysis	Data analysis was mainly performed using the SciPy statistics module in Python (https://docs.scipy.org/doc/scipy/reference/stats.html). PyPhi was further used for analysis of network properties. The code developed for this analysis with the PyPhi module has been uploaded and made available with open access on ZENODO (https://zenodo.org/record/8033892).<br><br>Image processing: For obtaining resting-state networks consisting of 5 regions, we developed a pipeline that merges the parcellation scheme presented by Gorden et al, 2016 (Petersen Neuroimaging Lab, source code: https://sites.wustl.edu/petersenschlaggarlab/resources/), along with a k-means algorithm to group parcels into the representative regions. Further image processing employed several other softwares: SPM (http:www.fil.ion.ucl.ac.uk/spm), FSL (https://fsl.fmrib.ox.ac.uk/fsl/fslwiki/), SimpleITK (http://www.simpleitk.org/) and Dipy (http://nipy.org/dipy/), RapidArt (https://www.nitrc.org/projects/rapidart/).

For manuscripts utilizing custom algorithms or software that are central to the research but not yet described in published literature, software must be made available to editors and reviewers. We strongly encourage code deposition in a community repository (e.g. GitHub). See the Nature Portfolio guidelines for submitting code & software for further information.

Data

Policy information about availability of data

All manuscripts must include a data availability statement. This statement should provide the following information, where applicable:

- Accession codes, unique identifiers, or web links for publicly available datasets
- A description of any restrictions on data availability
- For clinical datasets or third party data, please ensure that the statement adheres to our policy

Data was used in a previous paper entitled "Modeling an auditory stimulated brain under altered states of consciousness using the generalized ising model". This data is currently available in Openneuro.org (https://openneuro.org/datasets/ds003171). The processed time-series use to compute phi were uploaded as part of the supplementary material.

Field-specific reporting

Please select the one below that is the best fit for your research. If you are not sure, read the appropriate sections before making your selection.

☒ Life sciences ☐ Behavioural & social sciences ☐ Ecological, evolutionary & environmental sciences

For a reference copy of the document with all sections, see nature.com/documents/nr-reporting-summary-flat.pdf

Life sciences study design

All studies must disclose on these points even when the disclosure is negative.

Sample size	The data obtained for this study recruited 17 healthy subjects for acquisition with fMRI and propofol. This number of subject is sufficiently high considering the administration of an anesthetic, which may involve some level of concern to subjects. For each subject, we obtained a collection of 11 resting-state networks, which were chosen to allow for analysis of a diverse range brain functions and activity patterns.
Data exclusions	No data was excluded from this study.
Replication	The results we obtained for integrated information were only applied to the sample of subjects that participated in this study. Although we have not applied this procedure to other neurological data, our procedure and its corresponding code can be applied to any other dataset.
Randomization	The entire population of subjects was used as a single group in this analysis (no splitting into groups).
Blinding	No blinding was necessary for the nature of this study, as each subject underwent an identical procedure with propofol and fMRI.

Reporting for specific materials, systems and methods

We require information from authors about some types of materials, experimental systems and methods used in many studies. Here, indicate whether each material, system or method listed is relevant to your study. If you are not sure if a list item applies to your research, read the appropriate section before selecting a response.

Materials & experimental systems

n/a	Involved in the study	
☒	☐	Antibodies
☒	☐	Eukaryotic cell lines
☒	☐	Palaeontology and archaeology
☒	☐	Animals and other organisms
☐	☒	Human research participants
☒	☐	Clinical data
☒	☐	Dual use research of concern

Methods

n/a	Involved in the study	
☒	☐	ChIP-seq
☒	☐	Flow cytometry
☐	☒	MRI-based neuroimaging

Human research participants

Policy information about studies involving human research participants

Population characteristics	17 healthy volunteers (4 women; mean age 24 years, SD = 5) participated in this study. All were native English speakers, right-handed, and had no history of neurological disorders.
Recruitment	Recruitment was achieved by posting printed advertisements throughout the university and sharing the study through word of mouth. We remunerated volunteers for their time and willingness to participate.

Ethics oversight

Ethical approval was obtained from the Health Sciences Research Ethics Board and Psychology Research Ethics Board of Western University (REB #104755).

Note that full information on the approval of the study protocol must also be provided in the manuscript.

Magnetic resonance imaging

Experimental design

Design type

Resting state fMRI

Design specifications

No block design structure. Four different acquisition types; one for each anesthetic condition (awake, mild sedation, deep sedation, recovery). Each of these included 245 TR (1 TR = 2 seconds).

Behavioral performance measures

N/A (resting - state)

Acquisition

Imaging type(s)

Primarily functional, but structural images also obtained for co-registration and parcellation.

Field strength

3 T

Sequence & imaging parameters

Echo-planar sequencing was used to acquire functional images with the following properties: 33 slices, voxel size: 3 × 3 × 3 mm3, inter-slice gap of 25%, TR = 2000 ms, TE = 30 ms, matrix size = 64 × 64, FA = 75°. Resting-state scans had 256 vol. We also obtained an anatomical scan using a T1-weighted 3D MPRAGE (Magnetization Prepared - RApid Gradient Echo) sequence with the following properties: 32 channel coil, voxel size: 1 × 1 × 1 mm3, TE = 4.25 ms, matrix size = 240 × 256 × 192, FA = 9°.

Area of acquisition

Whole brain scan

Diffusion MRI ☐ Used ☒ Not used

Preprocessing

Preprocessing software

T1 images were preprocessed using the following toolboxes: SPM, FSL, SimpleITK, and Dipy. Preprocessing for T1- weighted imaging consisted of the following: manual removal of the neck, removal of non-brain tissue using the FMRIB Software Library (FSL), correction of non-uniformity in low frequency intensity based on the N4 bias field correction algorithm (obtained from SimpleITK), image denoising with the nonlocal means algorithm from Dipy, and spatial normalization to standard stereotactic Montreal Neurological Institute (MNI) space using the SPM12 normalization algorithm. Head motion and slice timing correction were performed using the MCFLIRT algorithm from FSL. We then ran artifact outlier detection, followed by artifact correction using RapidArt. Spatial smoothing was applied to the fMRI data using a Gaussian kernel (8 mm full width at half maximum as implemented in SPM12).

Normalization

fMRI data were co-registered onto the T1 image and spatially normalized to the MNI space with the SPM12 normalization algorithm.

Normalization template

standard stereotactic Montreal Neurological Institute (MNI) space

Noise and artifact removal

The fMRI time-series were then cleaned by removing spurious variance by means of nuisance signal regression, which was based on the average time series of external regions of noninterest (white matter and cerebrospinal fluid). A rigid body transformation, which was obtained from head-motion correction with FSL, yielded six motion parameters for translation and rotation in three dimensions, which were also included in the nuisance regressors. The time-series were then detrended and filtered using a bandpass Butterworth filter with cut-off frequencies set at 0.01 Hz and 0.1 Hz.

Volume censoring

The three initial volumes were discarded to avoid T1 saturation effects in the fMRI data

Statistical modeling & inference

Model type and settings

ICA used to extract networks and pearson correlation used to obtain correlations that defined each network's 5 regions of interest.

Effect(s) tested

N/A

Specify type of analysis: ☒ Whole brain ☐ ROI-based ☐ Both

Statistic type for inference
(See Eklund et al. 2016)

N/A

Correction

N/A

Models & analysis

n/a	Involved in the study
☐	☒ Functional and/or effective connectivity
☒	☐ Graph analysis
☒	☐ Multivariate modeling or predictive analysis

Functional and/or effective connectivity

Pearson correlation used for reference, the principal metric used to analyze the networks was integrated information.
